# Rh single atoms on TiO_2_ dynamically respond to reaction conditions by adapting their site

**DOI:** 10.1038/s41467-019-12461-6

**Published:** 2019-10-03

**Authors:** Yan Tang, Chithra Asokan, Mingjie Xu, George W. Graham, Xiaoqing Pan, Phillip Christopher, Jun Li, Philippe Sautet

**Affiliations:** 1 Department of Chemistry and Key Laboratory of Organic Optoelectronics and Molecular Engineering of the Ministry of Education, Tsinghua University, 100084 Beijing, China; 2 Department of Chemical and Biomolecular Engineering, University of California, Los Angeles, Los Angeles, CA 90095 USA; 3 Department of Chemistry and Biochemistry, University of California, Los Angeles, Los Angeles, CA 90095 USA; 4 California NanoSystems Institute, University of California, Los Angeles, Los Angeles, CA 90095 USA; 5 Department of Chemical Engineering, University of California, Santa Barbara, Santa Barbara, CA 93106 USA; 6 Department of Materials Science and Engineering, University of California Irvine, Irvine, CA 92697 USA; 7 Department of Materials Science and Engineering, University of Michigan, Ann Arbor, MI 48109 USA; 8 Department of Physics and Astronomy, University of California Irvine, Irvine, CA 92697 USA; 9 Irvine Materials Research Institute (IMRI), University of California Irvine, Irvine, CA 92697 USA; 10 Department of Chemistry, Southern University of Science and Technology, 518055 Shenzhen, China

**Keywords:** Catalytic mechanisms, Heterogeneous catalysis, Computational chemistry

## Abstract

Single-atom catalysts are widely investigated heterogeneous catalysts; however, the identification of the local environment of single atoms under experimental conditions, as well as operando characterization of their structural changes during catalytic reactions are still challenging. Here, the preferred local coordination of Rh single atoms is investigated on TiO_2_ during calcination in O_2_, reduction in H_2_, CO adsorption, and reverse water gas shift (RWGS) reaction conditions. Theoretical and experimental studies clearly demonstrate that Rh single atoms adapt their local coordination and reactivity in response to various redox conditions. Single-atom catalysts hence do not have static local coordinations, but can switch from inactive to active structure under reaction conditions, hence explaining some conflicting literature accounts. The combination of approaches also elucidates the structure of the catalytic active site during reverse water gas shift. This insight on the real nature of the active site is key for the design of high-performance catalysts.

## Abstract (web summary)

Single-atom catalysts are widely investigated heterogeneous catalysts; however, the identification of the local environment of single atoms under experimental conditions is still challenging. Here, the authors clearly demonstrate that Rh single atoms adapt their local coordination and reactivity in response to various redox conditions.

## Introduction

Supported noble metal nanoparticles are widely investigated heterogeneous catalysts due to their high activity and selectivity for valuable products. However, the high prices and finite resources of these noble metals limit their applications in large-scale production. Heterogeneous single-atom catalysts (SACs), which consist of isolated atoms anchored onto supports, maximize the efficiency of noble metal utilization^1–5^. Moreover, developing catalysts that precisely place the single metal atoms homogeneously at a single site on the support can offer high selectivity towards a specific product. Thus, as a bridge between homogeneous and heterogeneous catalysis, SACs have become a new frontier in catalysis science and thus attracted significant attention recently^6–15^.

A single atom (SA) on a support possesses unique chemical and physical properties due to its particular local chemical environment, inducing an electronic structure that differs from that of conventional supported nanoparticle catalysts. However, the identification of this local environment of SAs under experimental conditions, as well as operando characterization of structural changes of SAs during catalytic reactions, is still challenging despite the development of in situ microscopy and spectroscopy techniques^16^. The local environment determines the electronic structure, charge distribution, and oxidation state of the SAs, and hence also the stability, activity, and selectivity of the catalyst. Two main types of structures have been described for SACs: either substituting a cation at the oxide surface or supported on-top of the support, and these two types of SAs generally exhibit different catalytic properties^17^. Camellone and Fabris^18^ reported that substitutional Au^3+^ ions at the ceria surface, and not supported Au^+^ adatoms, activate molecular CO and oxidize it to CO_2_^18^. In the case of cobalt oxide (CoO)-supported Rh SACs, Rh substituted at the Co site was shown to be active and selective towards propene hydroformylation^19^, while Rh substituted at the O site (Rh_1_Co_3_ bimetallic single-cluster sites) exhibits an excellent catalytic performance in the reduction of NO with CO at low temperature^20^ and a potential application in ammonia synthesis via N_2_ reduction^21^. Datye and co-workers^22^ highlighted that atomically dispersed Pt on ceria can achieve a high activity for low-temperature CO oxidation after a steam treatment at 750 °C, which affects the environment of the single Pt atom on the support.

There are several factors that can potentially affect the local structure of SACs, including specific pretreatment, reactants, intermediate species, and temperature. Here, we consider a single Rh atom supported on the rutile TiO_2_(110) surface to investigate the stability of different Rh structures under typical experimental conditions with first-principles atomistic thermodynamics, in order to show how SAs dynamically respond to reaction conditions. Stabilization of SAC requires strong interaction between the metal atom and the oxide, and although several oxides have been shown to be adequate (Fe_3_O_4_, TiO_2_, CeO_2_, Co_3_O_4_), TiO_2_ avoids additional theoretical challenges linked with magnetic properties or relativistic effects. We experimentally characterize single Rh atoms on rutile after different reduction steps under hydrogen by CO probe molecule infrared (IR) spectroscopy and scanning transmission electron microscopy (STEM) imaging^23,24^. The combination clearly demonstrates that Rh SAs change their structure and adapt their catalytic site under reaction conditions towards a structure active for CO_2_ reduction to CO under hydrogen by reverse water gas shift (RWGS).

## Results

### Compared stability of various sites

**Fig. 1:**
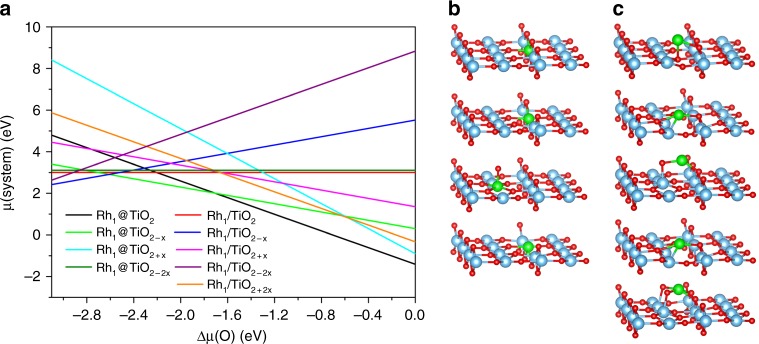
Stability and structure of substitutional (@) and supported (/) single-atom Rh. The TiO_2_(110) surface is considered, including O vacancies or adatoms. **a** Relative stability as a function of oxygen chemical potential Δ*μ*(O). **b**, **c** The optimal structures for substitutional (**b**) and supported (**c**) Rh SAs on the considered TiO_2_ surfaces. The structures in **b**, **c** follows the placement and order in the legend of **a**. Color code: O—red; Ti—blue; Rh—green

The two main parameters controlling the structure of the Rh atom/TiO_2_(110) interface are the oxygen stoichiometry for the TiO_2_ surface and the position of the Rh atom. We systematically studied the stoichiometric termination, two oxygen-deficient ones (0.65 and 1.30 vacancies by nm^2^) and one oxygen-rich one (0.65 additional O by nm^2^), and we explored a large range of positions for the Rh atom, in substitution of a Ti atom (denoted as Rh_1_@support) or supported on the surface (denoted as Rh_1_/support), and of relative positions between the Rh atom and the vacancies/additional O. This allowed us to generate multiple environments and electronic structures for the Rh atom. The stability of the various terminations can be compared using first-principles atomistic thermodynamics, using oxygen chemical potential (*μ*) as a descriptor (Ti chemical potential is then fixed from the bulk energy of TiO_2_). For each selected TiO_2_ surface termination, and each type of Rh site (supported or substitutional), all Rh atom positions have been evaluated, but only the most stable one is considered in the surface stability diagram (Fig. 1). Other positions and their relative energies can be seen in Supplementary Figs. 1 and 2.

Under oxygen-rich conditions (high chemical potential of oxygen, −1.7 < Δ*μ*(O) < 0), Rh substituted at the 6-fold Ti site in the stoichiometric TiO_2_ (Rh_1_@TiO_2_) is the most favorable structure (Fig. 1a). Terminations with additional O atoms, and Rh appearing as a supported oxide moiety (Rh_1_@TiO_2 + *x*_, Rh_1_/TiO_2 + 2x_), are close in energy in the highest part of the interval, but never appear as most stable. Under oxygen-poor conditions, with a decreased *μ*(O), one oxygen vacancy is formed, and initially the Rh atom remains in the substitutional site (Rh_1_@TiO_2−*x*_), the vacancy corresponding to a surface O atom bridging Rh and Ti. If *μ*(O) is further decreased below −2.5 eV, the most stable configuration shifts from the substituted model (Rh_1_@TiO_2−x_) to the supported model (Rh_1_/TiO_2−*x*_). The most favorable structure for this adsorbed Rh atom is above an O vacancy corresponding to a 3-fold subsurface oxygen, which is not the most stable vacancy position on the bare TiO_2_ surface. Under typical conditions used during catalyst calcination (700 K in air, which is used to remove organic species deposited during catalyst synthesis), where Δ*μ*(O) is calculated to be −0.78 eV, the substitutional structure is most favorable and the surface is stoichiometric. The local environment for Rh_1_@TiO_2_ is Rh_1_O_6_ with a calculated Rh charge of +1.84 |*e*| and a formal oxidation number of Rh(+4). A supported neutral Rh atom on stoichiometric TiO_2_ (Rh_1_/TiO_2_) is ~3 eV less stable. Two additional O atoms can stabilize the supported Rh as a cation (RhO_2_/TiO_2_), but this structure remains ~1 eV less stable than the substitutional cation site. Changing the temperature and O_2_ partial pressure does not affect the preferred structure for Rh (Supplementary Fig. 3). Rh SAs always prefer to localize at the 6-fold Ti site under the presence of O_2_ gas even in cases of low O_2_ partial pressure or rather high temperature. The result agrees with high-angle annular dark-field-STEM (HAADF-STEM) images for various oxide-supported SACs, which show the metal at the site of the oxide cation, suggesting that it tends to replace the metal cations in the oxides^1,25^.

### Stability in H_2_ reduction conditions

Hydrogen can adsorb on the Rh atom or on the two-coordinated surface O atoms of TiO_2_, and the optimal coverage at thermodynamic equilibrium depends on the H chemical potential *μ*(H) (Supplementary Fig. 4). Under a typical condition for H_2_ reduction (0.1 atm H_2_ at 500 K), where Δ*μ*(H) is calculated to be −0.35 eV, a high coverage of H on the surface O atoms is reached with 7H in the selected unit cell (containing 8 two-coordinated O atoms) for Rh_1_@TiO_2_ and Rh_1_@TiO_2−*x*_, and 6H for the other structures (Rh_1_@TiO_2−2*x*_, Rh_1_/TiO_2_, Rh_1_/TiO_2−*x*_ and Rh_1_/TiO_2−2*x*_, respectively). The optimal hydrogenated structures are shown in Supplementary Fig. 5. Rh at a substitutional site is coordinated to six O atoms, and hence unable to bind H. O vacancies, created preferentially near the Rh, lead to unsaturation and 1 (resp. 2) H binds to the substituted Rh in the presence of 1 (resp. 2) vacancies. In a rather similar way, the supported Rh atom binds 2H atoms, if 1 or 2 O vacancies are present.

**Fig. 2:**
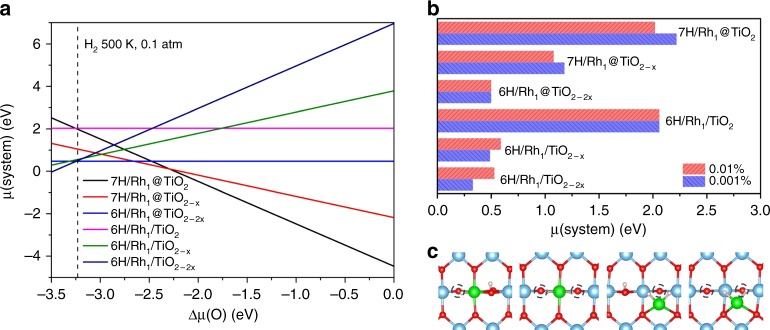
Stability of single Rh atom on TiO_2_(110) under a pressure of H_2_. Substitutional (@) and supported (/) sites are compared. **a** Relative stability as a function of Δ*μ*(O) under a typical condition of H_2_ reduction (H_2_ pressure of 0.1 atm at 500 K, i.e. Δ*μ*(H) = −0.35 eV). **b** Free energy for different Rh atom configurations in specific condition corresponding to water formation equilibrium (Δ*μ*(H) = −0.35 eV and Δ*μ*(O) = −3.23 (−3.33) eV for 0.01% (0.001%) conversion). The dashed line in **a** represents the condition (0.01% conversion) in **b**. **c** The local environment of Rh SAs in Rh_1_@TiO_2−*x*_, Rh_1_@TiO_2−2*x*_, Rh_1_/TiO_2−*x*_, and Rh_1_/TiO_2−2*x*_ (from left to right). Color code: O—red; Ti—blue; Rh—green. O vacancies are indicated by small dashed circles. Conversion fixes the pressure of H_2_O. For instance, 0.01% conversion means a H_2_O pressure of 10^−5^ atm. Then *μ*(O) is obtained by *μ*(H_2_O) − *μ*(H_2_)

At the given *μ*(H) value, the relative stability for each model still depends on the chemical potential of oxygen, as shown in Fig. 2a, in a similar way to Fig. 1a. A major difference is that the high pressure of hydrogen drives the system into an O-poor condition. Under reduction conditions, hydrogen will react with surface O atoms to form water and if we assume an H_2_ conversion of 0.01% to H_2_O, and still 0.1 atm H_2_ at 500 K, Δ*μ*(O) is then fixed at the low value of −3.23 eV. At this O chemical potential, the substitutional and supported Rh atom structures have almost the same free energy (Fig. 2b) and hence both coexist at thermodynamic equilibrium. This supported Rh becomes stable vs. the substitutional cation upon the formation of two O vacancies around Rh, and in this situation, it is liganded to two H atoms. Therefore, Rh SAs on TiO_2_ adapt their site from substitutional to supported when going from oxygen-rich preparation to H_2_-reduction conditions. Mobility of the TiO_2_ surface at high temperature under H_2_ allows filling of the Ti vacancy formed after extraction of the Rh atom^16^.

**Fig. 3:**
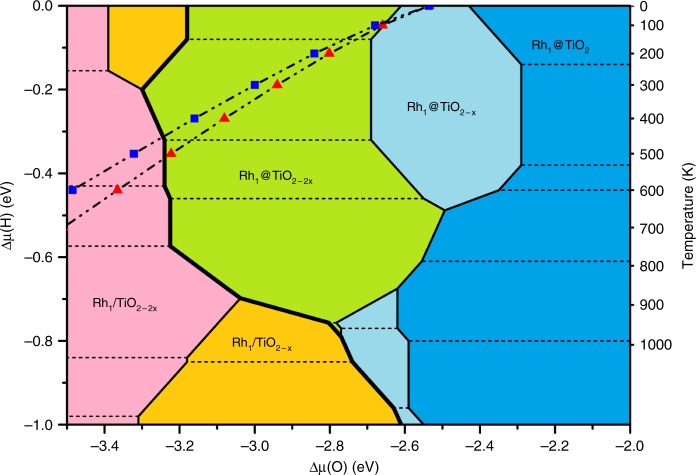
Surface stability diagram for Rh single atom on TiO_2_(110) in the presence of H_2_. Axes represent the H and O chemical potentials (noted ∆*µ*(H) and ∆*µ*(O) in eV). Different colors indicate the various configurations for the Rh and the TiO_2_ surface, where blue, light blue, and green denote regions where Rh is preferentially substituting a six-coordinated surface Ti with zero, one, or two O vacancies (Rh_1_@TiO_2_, Rh_1_@TiO_2 – *x*_, Rh_1_@TiO_2 −2*×*_) and orange and pink zones where the supported Rh structure is favored (Rh_1_/TiO_2−*x*_ and Rh_1_/TiO_2−2*×*_), respectively. The amount of hydrogen adsorbed on the TiO_2_ surface and the Rh atom depends on ∆*µ*(H) and dash lines limit the zones corresponding to different hydrogen coverage (see Supplementary Fig. 6). The red triangle (blue square) line shows the relation between Δ*μ*(O) and Δ*μ*(H) when water formation reaction is included with 0.01% (0.001%) conversion. Δ*μ*(H) values for 0.1 atm H_2_ and various temperatures are shown on the right vertical axis

If Δ*μ*(O) and Δ*μ*(H) are independent, a 2D surface stability diagram can be constructed as a function of these two descriptors (Fig. 3). High Δ*μ*(O) stabilizes the substitutional sites, and low Δ*μ*(O) the supported sites, but the frontier between the stability regions of the two sites (shown as a thick black line) is moved towards lower Δ*μ*(O) when Δ*μ*(H) (and hence the H coverage) is increased, especially in the temperature region corresponding to reduction conditions (400–600 K). This trend is explained by the strong adsorption energy of H on Rh (−2.30 eV) in the presence of two O vacancies, which extends the stability of the green zone in Fig. 3. Hence, hydrogen pressure stabilizes both the substitutional and the supported site, but the shift to very O-poor conditions strongly favors the supported site, which becomes more stable.

### Stability under a pressure of CO

**Fig. 4:**
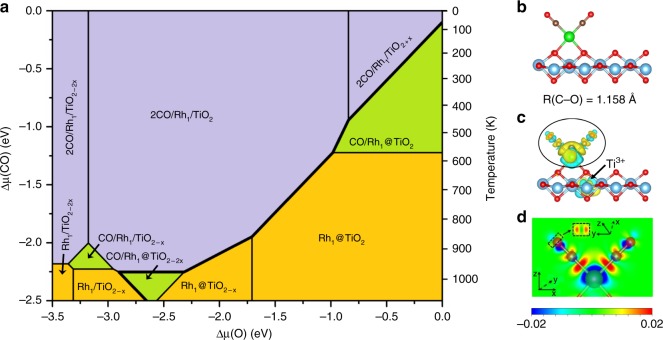
Structure and stability Of Rh single atom in the presence of CO. **a** Surface stability diagram for Rh single atom on TiO_2_(110) as a function of CO and O chemical potentials. Colors indicate the different number of CO molecules adsorbed on Rh_1_TiO_2_, where orange, green, and purple represent the 0CO, 1CO, and 2CO, respectively. The temperature in right axis corresponds to the Δ*μ*(CO) in left axis with 0.1 atom CO. **b** Local environment of Rh_1_/TiO_2_ in the presence of CO. **c**, **d** 3D (**c**) and 2D (**d**) representations of the charge density difference between 2CO/Rh_1_/TiO_2_ and Rh_1_/TiO_2_, show the donation and back-donation electron transfers

Upon submitting the system to a pressure of CO, the preferred structure is governed by the O and CO chemical potentials. CO adsorbs on the supported SA and all possible adsorption sites for SA Rh with one or two CO adsorbed have been tested (Supplementary Fig. 7). The most stable structure for each Rh site, TiO_2_ termination, and CO coverage is chosen to establish the stability diagram. The complete 2D stability diagram, as a function of Δ*μ*(O) and Δ*μ*(CO), is shown in Fig. 4a. Partial diagrams constrained to one CO or two CO adsorption for a fixed Δ*μ*(CO) value can be seen in Supplementary Fig. 8. Upon CO pressure, the zones corresponding to Rh being stable as a supported atom (purple in Fig. 4a) are strongly extended. This is especially the case for a typical condition of CO adsorption used in volumetric chemisorption or probe molecule IR experiments (a 10% gas mixture of CO, and 90% He at atmospheric pressure and room temperature), in which Δ*μ*(CO) is calculated to be −0.59 eV, a situation where the supported Rh is more stable than the substitutional one for the almost entire range of O chemical potential considered here (Fig. 4a and Supplementary Fig. 9). CO adsorption hence completely changes the thermodynamic site preference for the Rh atom, irrespective of the presence of O vacancies. This is explained by the fact that CO adsorbs significantly more strongly on the supported Rh (−4.89 eV for Rh_1_/TiO_2_), compared to the substitutional Rh (−2.74 eV for Rh_1_@TiO_2−2*x*_). Charge density difference demonstrates that the strong interaction between Rh SAs and CO molecules results from not only Rh-C σ-bonding but also back-donation from the metal towards the antibonding π*_CO_ orbital (Fig. 4c, d).

Hence thermodynamics under CO pressure tends to drive all Rh atoms to a supported site, if the temperature is high enough for kinetic aspects not to be limiting. Thus, the CO molecule plays a role in stabilizing the supported Rh SAs. Experimentally, CO is typically adsorbed on Rh after H_2_ reduction, so the surface is partially reduced by H_2_ with the formation of two oxygen vacancies and thus the most favorable structure under CO pressure should be 2CO/Rh_1_/TiO_2−2*x*_.

To further illustrate the potential for Rh site modification of hydrogen or CO adsorption, we can define the driving energy of the transformation as the difference of free energy between the most favorable supported site and the most favorable substituted site, a negative driving energy indicating that the site change towards the supported case is thermodynamically favorable. During CO adsorption, the driving energy is markedly negative, with a value of ~−1.5 eV in the relevant region of chemical potential (see Supplementary Fig. 10b). This driving energy for site change is much stronger under a pressure of CO than under a pressure of H_2_, where it becomes negative only at very low *μ*(O) and remains close to energy neutral. This result further proves that CO and H_2_ molecules adapt the sites of SAs via different mechanisms and with different strength due to difference in their bonding mechanism.

### Stability vs. cluster formation and FTIR studies

Thermodynamic analyses hence show that Rh SACs on TiO_2_ will adapt their sites in response to reaction conditions, such as under H_2_ or CO pressure. Beyond its position on the oxide, the stability of the SA vs. clustering is another key aspect, especially when it is in supported situation and hence has potential mobility. Our calculations show that under a pressure of CO (2CO/Rh/TiO_2_ structure), dimerization of the Rh(CO)_2_ species is less stable by 1.14 eV (Supplementary Fig. 11). Hence initial clustering of the SA is thermodynamically unfavorable. In addition, the Rh SA liganded to two CO is more stable than bulk Rh metal (Supplementary Fig. 9), precluding the formation of large nanoparticles. Similarly, under H_2_ reduction condition (6H/Rh/TiO_2−*x*_ structure), the dimerization of the RhH_2_ surface species into a supported Rh_2_H_4_ unit is endothermic by 1.24 eV (Supplementary Fig. 11). However, in that case, the H-liganded Rh SA is less stable than bulk Rh (Fig. 2c). Since dimer formation is endothermic, the nucleation process is difficult, and the SA is metastable at reasonable temperature. There is hence a risk of particle formation only under H_2_ at high temperature.

**Fig. 5:**
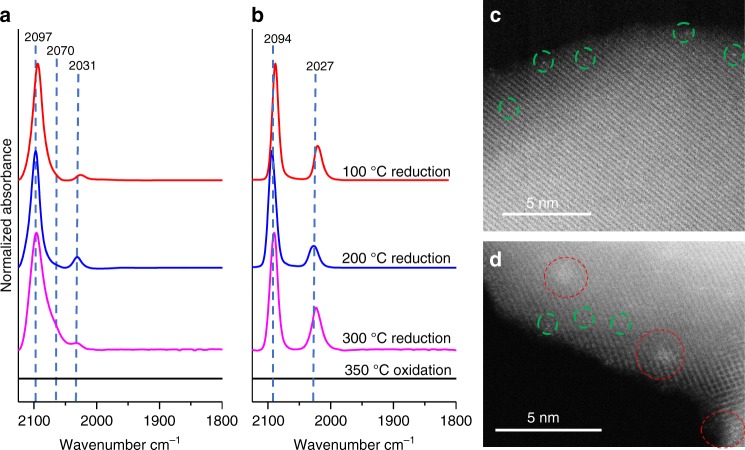
Rh SAC on TiO_2_ following varying environmental treatments. IR spectra of CO adsorbed at full saturation coverage following varied pretreatment conditions (oxidized at 350 °C (black), reduced at 100 °C (red), 200 °C (blue) and 300 °C (pink)) measured at **a** −120 °C and **b** 20 °C. Corresponding representative high-angle annular dark-field (HAADF) STEM images of **c** 100 °C H_2_ reduction case in which single Rh atoms are observed and **d** 300 °C H_2_ reduction case in which small Rh clusters and Rh single atoms are observed. Green circles identify the single Rh atoms, while red circles identify Rh clusters

In order to validate our conclusions, we synthesized Rh SAs on rutile TiO_2_ support, varied in situ pretreatments conditions, and used spectroscopy and microscopy to analyze the evolution of the Rh structure and TiO_2_ support. CO probe molecule IR experiments were executed at −120 °C, which allows us to kinetically trap the structure, avoiding any changes induced by the adsorption of CO. CO adsorption on Rh SAs supported on TiO_2_ has previously been shown to yield a specific doublet of peaks at ∼2097 and ∼2028 cm^−1^, associated with the symmetric and asymmetric stretches of the Rh(CO)_2_ *gem-*dicarbonyl species^26^. Figure 5a shows the IR spectrum of CO adsorption at −120 °C on four differently in situ pretreated samples (oxidized at 350 °C, reduced at 100, 200, and 300 °C), to correlate with the predicted structures that would form in varying oxidation and reduction conditions. The pre-oxidized (350 °C in O_2_ for 1 h) sample does not adsorb CO at −120 °C. This is fully consistent with aforementioned calculation results, since during O_2_ pretreatment, O chemical potential is high (−0.61 eV) and Rh SAs are stabilized in a +4 oxidation state in the substitutional site of Ti (Rh_1_@TiO_2_), a situation where CO cannot adsorb on the Rh cation.

After reduction in H_2_ at 100 °C, CO adsorbs on Rh at −120 °C with a predominant stretch at 2094 cm^−1^ and a very small accompanying stretch at 2026 cm^−1^. The formation of a majority species with CO adsorbing to Rh atoms with a 1:1 stoichiometry agrees with calculations of the Rh@TiO_2−*x*_ species, which show preference for the adsorption of single CO with a vibrational frequency of 2071 cm^−1^. This is consistent with the pathway followed in Fig. 3, where Rh@TiO_2−*x*_ forms due to exposure of Rh@TiO_2_ species (that do not adsorb CO) to mild reduction conditions. Further reduction in H_2_ at 200 °C induces the growth of the stretch at 2031 cm^−1^ and a shift in the stretch at 2094 to 2097 cm^−1^. This result is consistent with the calculations that predict the formation of the *gem*-dicarbonyl species 2CO/Rh_1_/TiO_2−2*x*_ (Fig. 4b), and the calculated frequencies for CO are 2082 and 2023 cm^−1^, in good agreement with the experimental values. However, when the reduction temperature is increased to 300 °C, one notes the appearance of a CO band at 2070 cm^−1^, showing the agglomeration of Rh SAs in Rh clusters.

The primary conclusions of the CO probe molecule IR characterization were substantiated via ex situ atomic resolution STEM imaging of the Rh/TiO_2_ sample following reduction in H_2_ at 100 and 300 °C, see Fig. 5c, d and Supplementary Fig. 12. For the sample that had been reduced at 100 °C, single Rh atoms were primarily observed in all images. Alternatively, for the sample that had been reduced at 300 °C, a mixture of Rh clusters and SAs were observed. Both observations are in agreement with the interpretation of the CO IR characterization. It is interesting to note that while 300 °C reducing conditions in H_2_ were observed to drive encapsulation of Rh particles by reduced TiO_2_ overlayers (see Supplementary Fig. 13), the combination of CO IR and STEM imaging presented here suggests that single Rh atoms will not be encapsulated by TiO_2_ under similar conditions^27^.

When CO adsorption is performed at 20 °C, all three reduced samples show similar Rh(CO)_2_ spectral features, suggesting that CO adsorption, when the temperature is high enough to allow atom mobility, drives Rh atoms to similar supported Rh(CO)_2_ geometries, even though they may be in different starting locations, Fig. 5b. The small difference in the CO stretch frequencies likely derives from small differences in surface O-vacancy coverage on the TiO_2_ support. From the sample pretreated at 300 °C, the feature at 2070 cm^−1^ disappears after CO adsorption at 20 °C, showing that supported Rh particles can be readily fragmented to Rh SAs in the presence of CO, see Fig. 5b, in line with the calculated high stability of supported SAs in these conditions^28^. This scenario is reminiscent of the dynamic SA formation during nanogold catalysis^29–31^. As expected, the intensity of the symmetric stretch (~2090 cm^−1^) at 20 °C is stronger than that of the asymmetric stretch (2023 cm^−1^) for the Rh(CO)_2_ species due to the existence of nearby O vacancies in TiO_2_, but the exact ratio of intensity depends on reduction conditions.

It should be noted that for the pre-oxidized Rh sample, exposure to 20 °C CO does not change the properties at all, Fig. 5b, and in fact exposure to CO at 300 °C is required to induce O-vacancy formation that enables CO adsorption, see Supplementary Fig. 14. That is because the stable and highly coordinated substitutional site cannot provide an adsorption site for CO, so that CO is unable to interact and to transform the site to the supported structure. Hydrogen hence has a special role in creating O vacancies around the substitutional Rh cation, reducing its coordination and opening a channel for CO adsorption, and subsequent migration of the CO stabilized Rh species. The creation of oxygen vacancies when CO acts alone as a reductant at 300 °C is further evident because CO_2_ was observed to form, presumably through oxidation by TiO_2_ lattice oxygen, and these O vacancies in turn enabled the formation of Rh(CO)_2_ species (Supplementary Fig. 14).

X-ray photoelectron (XPS) spectra showed a downshift of the XPS Ti 2*p* core level by 0.3 eV, from 459.0 to 458.7 eV, when comparing oxidation at 350 °C to reduction under hydrogen at 200 °C, indicating a reduction of some of the Ti in the sample, most likely at the surface of the oxide nanoparticle as indicated by the DFT calculations. Additionally, the O1*s* level in the reduced case is also downshifted by 0.4 eV, together with the appearance of a new feature at higher (+1.9 eV) binding energy (Supplementary Fig. 15)^32,33^. This peak is assigned to the formation of surface hydroxyls under hydrogen pressure. In addition, by using the integrated area of the Ti and O peaks and associated sensitivity factors, it was observed that the O:Ti ratio decreased by 5% after reduction^34^. The XPS and IR results are in agreement with the DFT calculations, and that reduction of the Rh/TiO_2_ catalysts induces hydroxylation and oxygen vacancy formation, which in turn drive the switch in the preferred Rh local coordination, enabling interaction with adsorbates.

### Catalytic reactivity

In a nutshell, the combination of theory and experiment shows here that Rh atoms adapt their binding site to the presence of various reactants (O_2_, H_2_, and CO). It is hence important to explore whether this adaptive character has an impact on catalytic properties. We selected the RWGS reaction as an example for this purpose since when submitting a mixture of CO_2_ and H_2_ to a Rh on TiO_2_ catalyst, it was shown that dispersed Rh SAs are active and selective for CO formation (from RWGS), while Rh nanoparticles lead to methanation^35^.

**Fig. 6:**
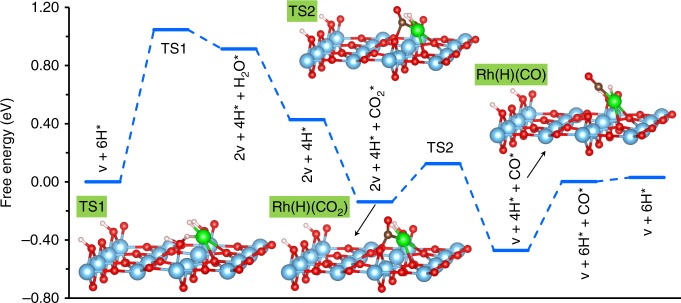
Free energy profile of the RWGS reaction on the Rh single atom. The supported structure of the Rh atom 6H/Rh_1_/TiO_2−*x*_ and the dissociation mechanism are considered. The first steps correspond to water formation and additional O-vacancy creation, followed by easy activation of CO_2_ forming CO. Color code: O—red; Ti—blue; Rh—green. *T* = 500 K and pressure in experimental conditions at equilibrium are used to evaluate free energies (Supplementary Table 1). The rate controlling intermediate is the supported Rh(H)(CO) unit. Atom color code: O—red; Ti—blue; Rh—green

Under reaction condition (500 K, 10 CO_2_:1 H_2_), 6H/Rh_1_@TiO_2−2*x*_ and 6H/Rh_1_/TiO_2−*x*_ are the most favorable hydrogenated structures for substitutional and supported models, respectively (Supplementary Table 1). Hence, both structures were considered as initial state for the RWGS pathways and considering two mechanisms: via CO_2_ dissociation or COOH-mediated. In the first case, the key step is the direct dissociation of CO_2_ to CO, while for the second possibility, adsorbed CO_2_ will react with surface OH species to form COOH that dissociates to OH and CO species^36^. For the supported Rh atom structure 6H/Rh_1_/TiO_2−*x*_, the CO_2_ dissociation path is the most favorable and the first step consists in forming a second O vacancy, necessary to activate CO_2_. One of the two H on Rh reacts with a neighboring OH species, forming one water and one oxygen vacancy, with a barrier of 1.05 eV (Fig. 6 and Supplementary Table 2). Water easily desorbs at reaction temperature, creating a vacancy site where CO_2_ can adsorb in an exergonic way, with an adsorption internal energy of −1.70 eV and a bent geometry, indicating a strong activation of CO_2_ at this site. Then, the dissociation of CO_2_ to CO occurs with a very low activation barrier (0.26 eV), the CO naturally coordinating to the Rh atom. The final steps (H_2_ dissociative adsorption and CO desorption) occur easily, but the order is important since H_2_ adsorption assists CO desorption. According to the energetic span model^37^, the effective barrier of the reaction is 1.52 eV. In contrast, the reaction is much more difficult if one starts from Rh in substitution of Ti in 6H/Rh_1_@TiO_2 −2x_. The Rh atom is saturated by two H atoms and, in order to create an adsorption site for CO_2_, one H must be transferred to a nearby O atom (Supplementary Fig. 16, Supplementary Table 3). Reaction with a surface –OH species to form H_2_O is not allowed here, since it is endothermic (+1.49 eV) with a barrier of 3.00 eV. Transfer to subsurface oxygen is still not favorable, with an endothermic energy of +1.46 eV and a barrier of 2.30 eV. Activation of the Rh center for RWGS is hence not possible, and the substitutional structure for the Rh atom is not catalytically active, because the strong adsorption energy of hydrogen on Rh prevents CO_2_ adsorption. This indicates clearly that the active site for Rh SAC on TiO_2_ in the case of the RWGS reaction is the supported site rather than the substitutional site. Further electronic structure analysis (see Supplementary Fig. 17) shows that Rh, liganded to two H atoms, is slightly negatively charged (−0.06 |*e*|) in 6H/Rh_1_/TiO_2−*x*_ and that its oxidation state is zero. In the unit cell, four protons and one vacancy provide six electrons. Five Ti^3+^ are located at the surface of the unit cell (representing 31% of surface Ti cations) and one electron is shared on the RhH_2_ unit. After creation of another vacancy by desorption of H_2_O (2v + 4H* intermediate on Fig. 6), one more Ti^4+^ is reduced to Ti^3+^ on the surface and Rh gains further electronic population (charge: −0.50 |*e*|). This negatively charged Rh can transfer electron to CO_2_, and promote the adsorption and activation of CO_2_.

The steady-state nature of the catalyst in reaction conditions is given by the most stable intermediate along the profile. For the supported Rh on TiO_2_, this corresponds to the coadsorption of CO and H on Rh, with three additional H adsorbates and one O vacancy on the TiO_2_ unit cell (Rh(H)(CO) in Fig. 6). In reaction conditions, it is 0.21 eV (Supplementary Table 1) more stable than the best substitutional site, which in the same conditions bears two H on the Rh and four H adsorbates and two O vacancy on TiO_2_. This Rh(H)(CO) structure is supported experimentally by in situ Fourier-transform infrared spectroscopy (FTIR) measurements under the same reaction conditions where a linear CO bonded to Rh appears at 2049 cm^−1^, close to the calculated value 2044 cm^−1^ (Supplementary Fig. 18a). Post-reaction analysis showed that the Rh species retained its SA structure (Supplementary Fig. 18b). The catalytic activity (turn over frequency, TOF) was calculated from the pathway (and in the conditions) of Fig. 6 by using the energetic span approach^37^. The value obtained (0.0056 s^−1^) is in qualitative agreement with the measured TOF values in similar conditions (~0.01 s^−1^)^35^. Comparing the theoretical and experimental results obtained here for rutile TiO_2_ supported single Rh atoms with reactivity measurements made on P25 mixed-phase TiO_2_ supports is justified based on CO IR measurements that suggest single Rh species localized on rutile domains of the P25 TiO_2_ support (Supplementary Fig. 19). Hence, the experimental and theoretical analysis agrees on the most stable species under reaction conditions and the TOF. This strongly validates our conclusion that, during RWGS reaction, Rh atoms will tend to move from the substitutional site favored during catalyst synthesis toward the supported site, more stable but also catalytically active.

By a combination of first-principles modeling and experiment, we have shown that single Rh atoms on TiO_2_ could dynamically adapt their site to the reaction conditions under reduction condition. This finding is consistent with the stability analyses of SAs on oxide surface that can vary with the site and reaction conditions^29,38^. The allusion of statically anchoring for a stable single metal atom on an oxide support can be misleading under such circumstances. In oxidative conditions, Rh prefers to substitute the six-coordinated Ti atoms in the surface plane of the oxide. In contrast, under a pressure of H_2_ or CO, it prefers to be supported on-top on the oxide. The roles of H_2_ and CO are however distinct in this scenario. CO efficiently stabilizes the supported site when adsorbed on it, but is unable to interact with the initial saturated substitutional site. In contrast, the stabilization induced by hydrogen is weaker, but it can decrease the Rh cation coordination by creation of O vacancies and hence open a channel for CO adsorption. In addition, the single metal atom is not bare, but covered by H atoms and/or CO molecule(s) in catalytic conditions. FTIR measurements of CO adsorption on SA Rh following varied pretreatment confirms the site change trend obtained from the calculations, and show that the transformation is kinetically accessible.

This adaptive character of the SA can only be accessed by operando studies and can be crucial for its catalytic reactivity, as illustrated here with the RWGS reaction. The as-prepared SAs, placed in substitution of titanium, are inactive for this reaction even after reduction by hydrogen and vacancy creation around the Rh. Especially noteworthy is the finding that the reductive condition of the reaction promotes the change of the site from substitution toward the supported structure, a situation where the SA is covered by H and CO and becomes catalytically active. FTIR during reaction again confirms the result. The H_2_ reactant, further assisted by the CO product, hence in situ switches the inactive as-prepared catalyst precursor into a highly active species. The dual character of the SAC with an off-site and an on-site, switchable through specific chemical interactions, could also explain the controversy in the literature on the inactive or active character of SAC, for example, in the case of Pt for CO oxidation and water gas shift reaction^24,39,40^.

Restructuring under reaction conditions is not a privilege of SAs, but also occurs for catalytic clusters and nanoparticles, as shown by in situ studies^41^. A key difference is that with SAs, the support plays the primary role in driving the reconstruction. In the present case, we showed that the change in oxidation state and coordination environment of Rh is driven by OH formation and O-vacancy formation at the TiO_2_ surface. In contrast, the dynamical behavior of a metal cluster in reaction conditions, although affected by the state of the support, is primarily driven by the metal cluster thermodynamics.

Our findings have provided insights on the critical questions of stability of heterogeneously supported SAs under reactive condition and the dynamic position changes of these atoms in redox reactions. The approach, and the proposed link between the transformation of the support and the restructuring of the metal, can in principle be extended to other SAC with similar metals (Rh, Pt, Ni, etc.) on reducible oxides (Fe_3_O_4_, CoO, CeO_2_) in order to understand how the chemical nature of oxide and metal controls the restructuring process. The message from the present study is that as-prepared catalysts provide structures with the metal at high oxidation state occupying the oxide cation position, and that migration to a more reduced, supported and adequately liganded structure might be required for optimal catalytic activity. Our results provide a detailed understanding of the local structure of SAs on oxide support and their adaptive and dynamic character under realistic catalytic conditions, which are key insights that chemists should have in mind when designing single SACs.

## Methods

### DFT parameters

All the calculations were performed using periodic DFT methods implemented in the Vienna Ab initio simulation package (VASP) code^42,43^. The projector augmented wave^44^ method was used for the interaction between the atomic cores and valence electrons. The valence orbitals of Ti (3*p*, 4*s*, 3*d*), Rh (4*d*, 5*s*), O (2*s*, 2*p*), C (2*s*, 2*p*), and H (1*s*) were described by plane-wave basis sets with cutoff energies of 400 eV. The exchange-correlation energies were calculated by the generalized gradient approximation with the Perdew–Burke–Ernzerhof functional^45^. Spin polarization was considered using unrestricted Kohn–Sham formalism. To correct the strong electron-correlation properties of these oxides, DFT + U calculations^46,47^ were performed with *U* = 4.2 eV for Ti, which is taken from the literature^48,49^.

The force threshold on each relaxed ion was set as 0.02 eV/Å. Monkhorst–Pack (1 × 1 × 1) Γ-centered **k**-points grid sampling within the Brillouin zones were used because of the large size of the surface models. The location and energy of transition states were calculated with the climbing-image nudged elastic band and dimer methods^50,51^. Vibrational analysis was further used to confirm the transition states with only one imaginary frequency.

### Computational models

The rutile TiO_2_(110) surface was modeled as periodic slabs with 12 layers (four tri-layers), with a vacuum gap between slabs at ~15 Å. A 4 × 2 surface cell was utilized and the bottom three layers were kept fixed to their bulk position.

In this work, we calculated the *adsorption energies* according to the following equation: *E*_ads_ = *E*_(slab + adsorbate)_ − *E*_(slab)_ − *E*_(adsorbate)_, where *E*_(slab + adsorbate)_, *E*_(slab)_, and *E*_(adsorbate)_ are the calculated electronic energy of species adsorbed on the surface, the bare surface, and the gas-phase molecule, respectively. Similarly, the *desorption energy* is defined as *E*_des_ = *E*_(slab + desorbate)_ − *E*_(slab)_ − *E*_(desorbate)_ and the *reaction energy* is defined as Δ*E* = *E*_(products)_ − *E*_(reactants)_. The charge density differences were evaluated using the formula Δ*ρ* = *ρ*_A + B_ − *ρ*_A −_ *ρ*_B_, where *ρ*_*X*_ is the electron density of *X*. Atomic charges were computed using the atom-in-molecule scheme proposed by Bader^52^.

### Stability descriptor

The system chemical potential *μ*(system) was used to measure the stability of different models with a consistent reference:

1 $${\mathrm{\mu }}({\mathrm{system}}) = {E}({\mathrm{system}})-{n}({\mathrm{O}}){\mathrm{\mu }}({\mathrm{O}})-{n}\left( {{\mathrm{TiO}}_2} \right){\mathrm{\mu }}\left( {{\mathrm{TiO}}_2} \right)-\left( {{E}\left( {{\mathrm{TiO}}_2} \right) + {\mathrm{\mu }}\left( {{\mathrm{Rh}}} \right)} \right),$$

where *E*(system) is the VASP calculated energy of Rh_1_TiO_2_ structures, and *E*(TiO_2_) is the energy of the bare TiO_2_ surface. The *μ*(Rh) and *μ*(TiO_2_) represent the chemical potential of bulk Rh and TiO_2_, respectively, and *μ*(O) is the free energy of oxygen atom. Bulk solid chemical potentials are taken from optimized DFT structures. The *n* values are set to balance the stoichiometry relative to a standard Rh_1_TiO_2_ system, namely, the TiO_2_ surface slab and bulk Rh. The relative free energy is calculated for each system by subtracting the energy of this standard system.

The chemical potential of oxygen atom, *μ*(O), is calculated as a function of temperature *T* and oxygen partial pressure *P*, using

2 $${\mathrm{\mu (O)}} = 1/2\left[ {{E}\left( {{\mathrm{O}}_2} \right) + \Delta {\mathrm{\mu }}\left( {{\mathrm{O}}_2\left( {{T},{P}} \right)} \right)} \right],$$

where Δ*μ*(O) is the value of *μ*(O) when using the half of *E*(O_2_) as zero reference. Similar definitions are used to Δ*μ*(H) and Δ*μ*(CO).

When we consider the absorbed species, the chemical potential of system with adsorbed species *μ*(system-ads) can be estimated by

3 $${\mathrm{\mu}}\left( {{\mathrm{system}} {\mbox{-}} {\mathrm{ads}}} \right) = {\mathrm{\mu }}\left( {{\mathrm{system}}} \right) + E_{{\mathrm{ads}}} - \Delta {\mathrm{\mu }}_{{\mathrm{ads}}}\left( {T,P} \right).$$

For H_2_ reduction, it should be

4 $${\mathrm{\mu }}({\mathrm{system}} {\mbox{-}} {\mathrm{H}}) =	 \,\,{E}({\mathrm{system}} {\mbox{-}} {\mathrm{H}}) - {n}({\mathrm{O}}){\mathrm{\mu }}({\mathrm{O}}) - {n}({\mathrm{TiO}}_2){\mathrm{\mu }}({\mathrm{TiO}}_2) \\ 	- ({\mathrm{E}}({\mathrm{TiO}}_2) + {\mathrm{\mu }}({\mathrm{Rh}})) - {n}({\mathrm{H}}){\mathrm{\mu }}({\mathrm{H}}),$$

where *μ*(system) is the chemical potential of system without adsorbed species; *E*_ads_ is the adsorption energy for adsorbed species and *μ*_ads_(*T*,*P*) is the chemical potential of adsorbed species. In the case of H_2_ reduction, an additional variable *μ*(H) is introduced, and *μ*(O) is given by

5 $${\mathrm{\mu }}({\mathrm{O}}) = {\mathrm{\mu }}({\mathrm{H}}_2{\mathrm{O}}) - {\mathrm{\mu }}({\mathrm{H}}_2).$$

### Sample preparation

Rh SACs were synthesized by utilizing strong electrostatic adsorption principles at the low Rh weight loading of 0.05 wt%. The rutile TiO_2_ support (US Nano # US3520, 30 nm diameter, 99.9% purity) and precursor (Sigma-Aldrich # 206261, Rhodium(III) chloride hydrate) were diluted in water separately at ratio of 4:1 and according to a support surface loading of 260 m^2^/l based on a measured TiO_2_ surface area of 26 m^2^/g. NH_4_OH was added to the separate support and precursor solutions to target a pH of 8.25. The precursor solution was injected at a rate of 4 ml/min into the support solution while constantly stirring. Then, the solution was heated to 70 °C until water evaporated and sample was dried completely. The sample was kept overnight in a 100 °C in an oven and then ex situ calcined in 10% O_2_ in He for 4 h at 350 °C. XPS was done to indicate that no Cl remained on catalyst to influence local Rh structure (Supplementary Fig. 20).

### FTIR characterization

The catalyst was loaded in a Harrick low-temperature reaction chamber mounted inside a Thermo Scientific Praying Mantis diffuse reflectance adapter set in a Nicolet iS10 FTIR spectrometer with a Mercury Cadmium Telluride (MCT) detector and mass flow controllers (Teledyne Hastings) were used to control the gas flow rates across the reactor bed. Catalysts were pretreated in situ by either oxidation for 30 min at 350 °C in 10% O_2_ in He, or subsequent reduction for 1 h at a specified temperature (100, 200, and 300 °C) in 10% H_2_ in Ar. CO probe molecule IR was executed by decreasing the temperature following pretreatment in argon below −120 °C in vacuum, then exposing to 10% CO in Ar for 10 min, followed by purging in Ar for 10 min, and spectra collection. The temperature was then increased to room temperature and 10% CO in Ar was flown to the cell for 10 min and flushed with Ar prior to room temperature spectra collection.

### STEM characterization

The two samples were taken from the same batch of Rh SAC. Both samples were ex situ oxidized at 350 °C for 30 min in pure O_2_ and then one was reduced at 100 °C and one at 300 °C for 60 min in 10% H_2_ in He. Atomic resolution bright field (BF) and HAADF-STEM images were taken on JEOL Grand ARM 300CF TEM/STEM with double spherical aberration correctors operated at 300 kV with probe current of 23 pA and pixel time of 4 μs. BF image was taken with outer collection angle of 31 mrad and HAADF image was taken with 53–180 mrad collection angle. Samples for STEM measurements were prepared by dropping 60 μl nanoparticles dispersion in methanol on a lacey carbon-coated copper grid.

### XPS characterization

The two samples were taken from the same batch of Rh SAC. Both samples were oxidized at 350 °C for 30 min in pure O_2_ and then one was reduced at 200 °C for 60 min in 5% H_2_ in Ar. After samples were oxidized or reduced, they were vacuum sealed without exposure to air, transferred to a glovebox under inert gas, and mounted on copper tape in a sample holder for XPS analysis. The sample holder was directly transferred from the glovebox to the chamber of the XPS to prevent air and moisture exposure. XPS characterization was carried out under vacuum using a Kratos AXIS ULTRA DLD XPS system equipped with an Al Kα monochromated X-ray source and a 165 mm mean radius electron energy hemispherical analyzer. Binding energy calibrations were done with reference to the carbon 1*s* peak by adjusting spectra to 284.8 eV.

## Supplementary information

Supplementary Information

Peer Review File

## Data Availability

All data supporting the findings of this study are available within the Article and its Supplementary Information and/or from the corresponding authors upon reasonable request.
