# Supplementary material for: Rh single atoms on TiO2 dynamically respond to reaction conditions by adapting their site
Source: Nat Commun. 2019 Oct 3;10:4488. doi: 10.1038/s41467-019-12461-6 (PMC6776542; doi:10.1038/s41467-019-12461-6)
Supplement: Supplementary file 1 — Supplementary Information [file 41467_2019_12461_MOESM1_ESM.pdf]

Supporting information for
Rh single atoms on TiO_2 dynamically respond to reaction conditions by adapting their site

Yan Tang,[1,2,3,4] Chithra Asokan,[5] Mingjie Xu,[6] George W. Graham,[6,7] Xiaoqing Pan ,[6,8,9] Phillip Christopher,[5] Jun Li[1,10*] and Philippe Sautet[2,3,4*]

[1] Department of Chemistry and Key Laboratory of Organic Optoelectronics & Molecular Engineering of the Ministry of Education, Tsinghua University, Beijing 10084, China

[2] Department of Chemical and Biomolecular Engineering, University of California, Los Angeles, Los Angeles, California 90095, United States

[3] Department of Chemistry and Biochemistry, University of California, Los Angeles, Los Angeles, California 90095, United States

[4] California NanoSystems Institute, University of California, Los Angeles, Los Angeles, CA 90095, United States

[5] Department of Chemical Engineering, University of California, Santa Barbara, Santa Barbara, California 93106, United States

[6] Department of Materials Science and Engineering, University of California Irvine, Irvine, California 92697, USA.

[7] Department of Materials Science and Engineering, University of Michigan, Ann Arbor, Michigan 48109, USA.

[8] Department of Physics and Astronomy, University of California Irvine, Irvine, California 92697, USA.

[9] Irvine Materials Research Institute (IMRI), University of California Irvine, Irvine, California 92697, USA.

[10] Department of Chemistry, Southern University of Science and Technology, Shenzhen 518055, China

*e-mail: sautet@ucla.edu; junli@tsinghua.edu.cn

Supplementary Discussion

Free energy calculations: system chemical potential

The system chemical potential μ(system) is calculated, using Eq. 1, for each configuration from the following formulas:

$\mu(Rh_1/TiO_2) = E(Rh_1/TiO_2) - (E(TiO_2) + \mu(Rh))$ for supported single-atom Rh_1 on TiO_2 surface.

$\mu(Rh_1@TiO_2) = E(Rh_1@TiO_2) - 2\mu(O) + \mu(TiO_2) - (E(TiO_2) + \mu(Rh))$ for substituted single-atom Rh_1 on TiO_2 surface.

$\mu(Rh_1/TiO_{2+nx}) = E(Rh_1/TiO_{2+nx}) - n\mu(O) - (E(TiO_2) + \mu(Rh))$ for supported single-atom Rh_1 on oxidized TiO_2 surface, n means the number of extra O atoms.

$\mu(Rh_1@TiO_{2+nx}) = E(Rh_1@TiO_{2+nx}) - (n+2)\mu(O) + \mu(TiO_2) - (E(TiO_2) + \mu(Rh))$ for substituted single-atom Rh_1 on oxidized TiO_2 surface, n means the number of extra O atoms.

$\mu(Rh_1/TiO_{2-nx}) = E(Rh_1/TiO_{2-nx}) + n\mu(O) - (E(TiO_2) + \mu(Rh))$ for supported single-atom Rh_1 on reduced TiO_2 surface, n means the number of oxygen vacancies.

$\mu(Rh_1@TiO_{2-nx}) = E(Rh_1@TiO_{2-nx}) + (n-2)\mu(O) + \mu(TiO_2) - (E(TiO_2) + \mu(Rh))$ for supported single-atom Rh_1 on reduced TiO_2 surface, n means the number of oxygen vacancies.

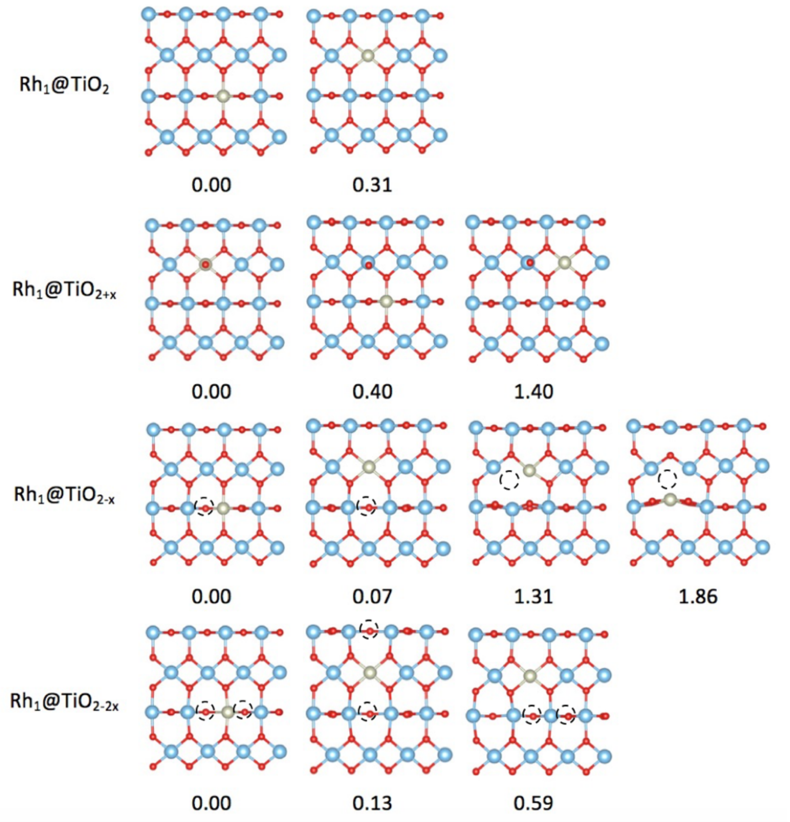

Supplementary Figure 1 | Possible adsorption sites for single-atom Rh in substituted models. The value under each configuration indicates its relative energy with respect to the most stable one. Color code: O-red; Ti-blue; Rh-gray. The dashed circle represents the oxygen vacancy site.

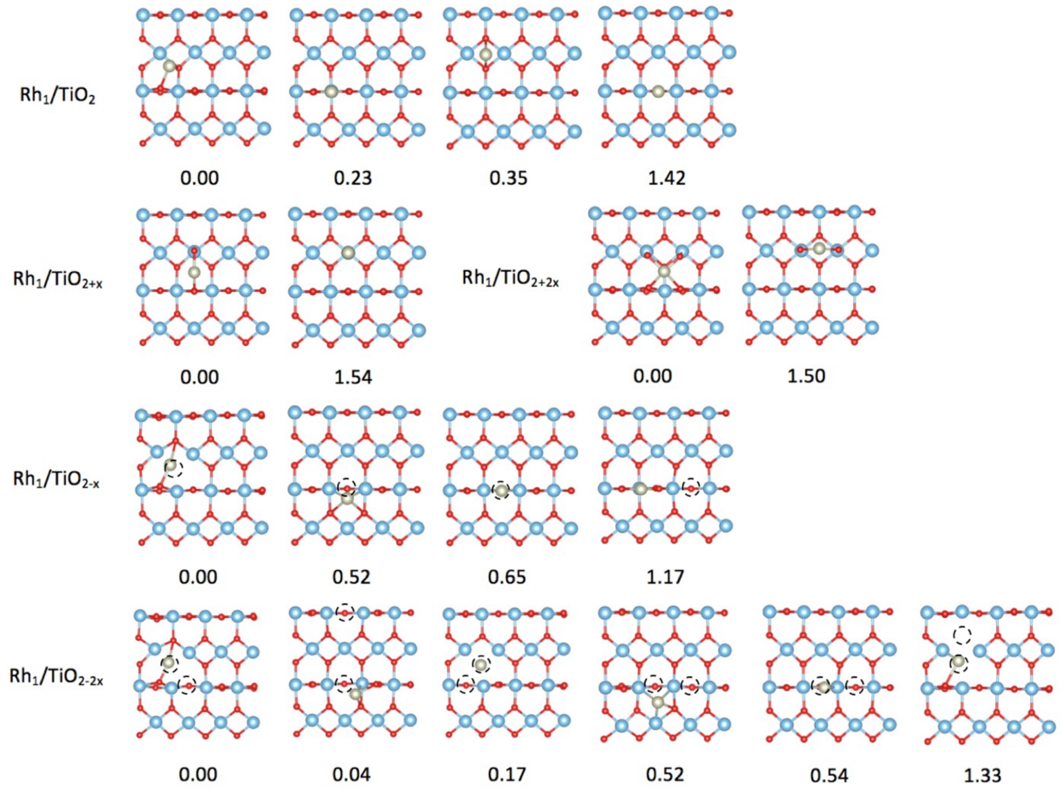

Supplementary Figure 2 | Possible adsorption sites for single-atom Rh in supported models. The value under each configuration indicates its relative energy with respect to the most stable one. Color code: O-red; Ti-blue; Rh-gray. The dashed circle represents the oxygen vacancy site.

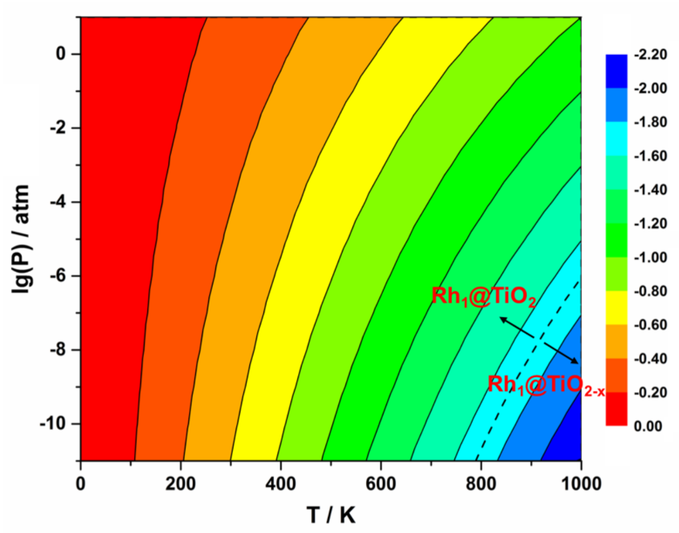

Supplementary Figure 3 | Phase diagram for most stable single-atom Rh states on TiO_2 (110) surface. The color represents the value of chemical potential of oxygen, which depends on pressure and temperature of oxygen molecule. The dashed line indicates the boundary line between Rh_1@TiO_2 and Rh_1@TiO_{2-x} (corresponding to the formation of one O vacancy in the surface).

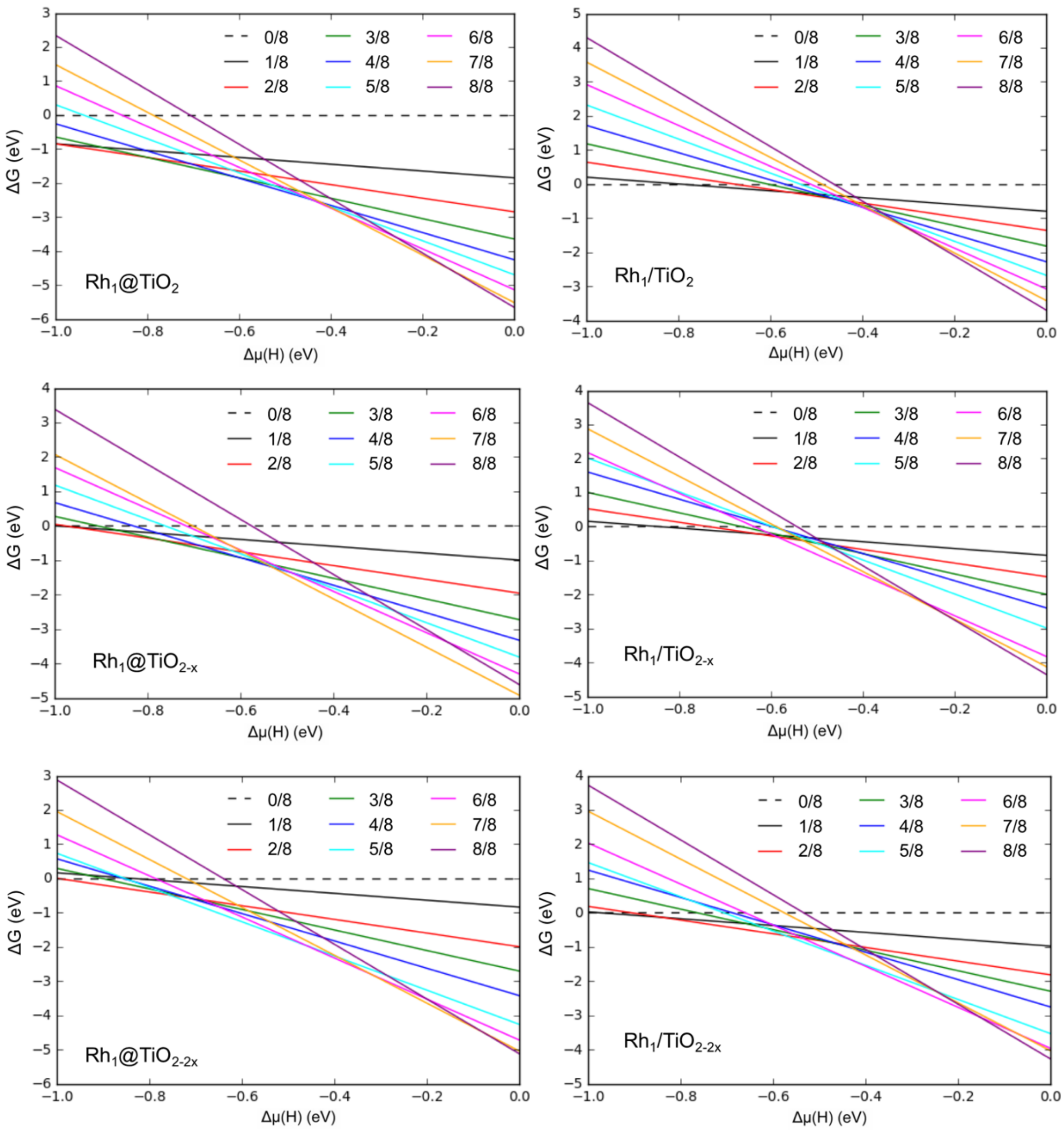

Supplementary Figure 4 | The energy change with different coverages of hydrogen on various Rh_1TiO_2 models as a function of Δμ(H). ΔG represents the free energy difference between the hydrogen-adsorbed system and the initial system without hydrogen atoms on the surface.

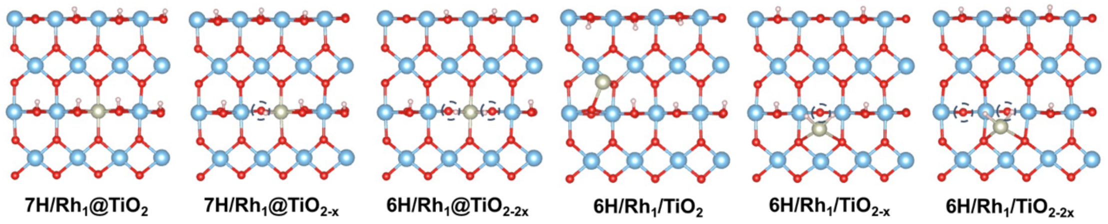

Supplementary Figure 5 | Most stable optimized configurations for hydrogen adsorbed on Rh_1SiO_2 SACs. Color code: O-red; Ti-blue; Rh-gray. The dashed circle represents the oxygen vacancy site.

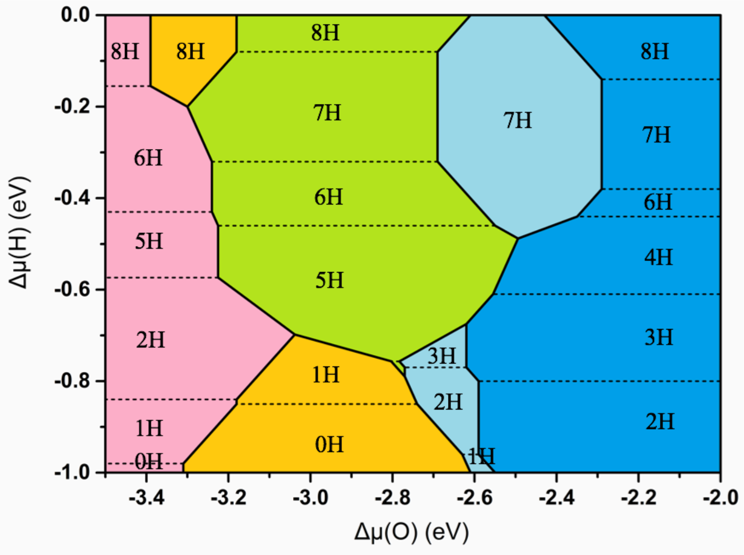

Supplementary Figure 6 | Surface stability diagram for single atom Rh on the TiO_2(110) surface in the presence of H_2 as a function of H and O chemical potentials (noted Δμ(H) and Δμ(O))**.** The amount of hydrogen adsorbed on the TiO_2 surface and the Rh atom depends on Δμ(H) and dash lines limit the zones corresponding to different hydrogen coverage. Different colors indicate the various configurations for the Rh and the TiO_2 surface, where blue, light blue, green, orange and pink regions denote $Rh_1@TiO_2$, $Rh_1@TiO_{2-x}$, $Rh_1@TiO_{2-2x}$, Rh_1/TiO_{2-x} and Rh_1/TiO_{2-2x}, respectively.

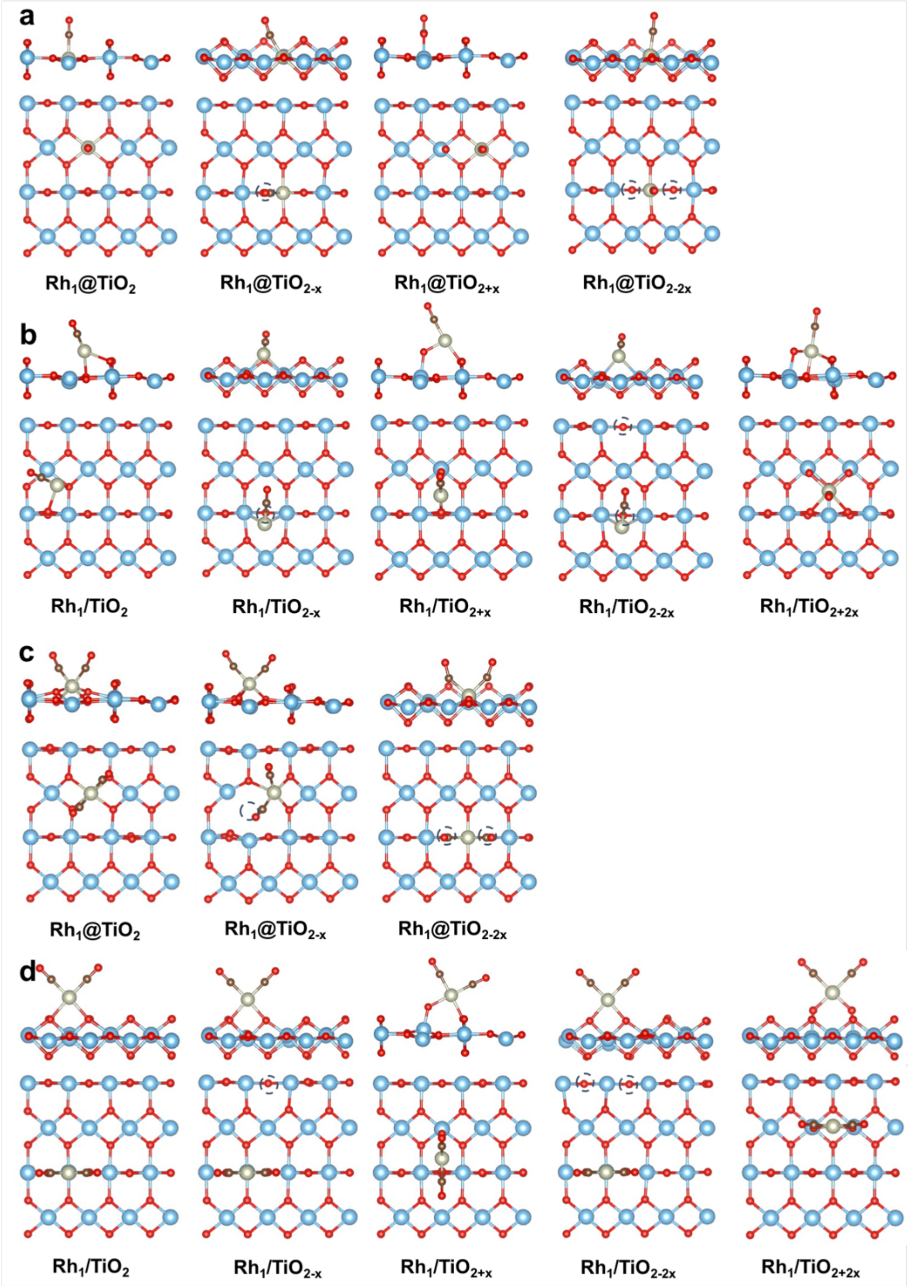

Supplementary Figure 7 | Optimized configurations for CO adsorbed on Rh_1SiO_2 SACs. a,b, one CO adsorption. c,d, 2CO adsorption. Color code: O-red; Ti-blue; Rh-gray. The dashed circle represents the oxygen vacancy site.

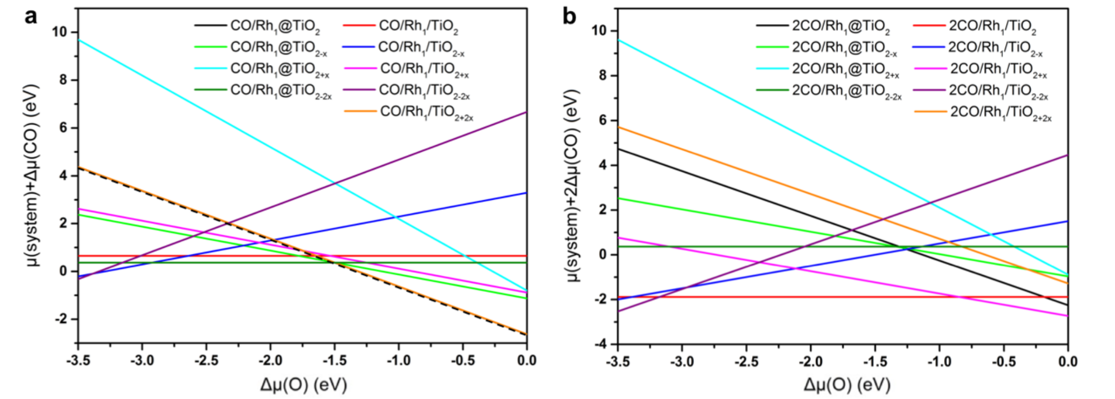

Supplementary Figure 8 | Relative stability of Rh_1TiO_2 with CO adsorbates. a. 1CO adsorption. The most stable site from O rich to O poor conditions (right to left on x axis) is CO/Rh_1@TiO_2 (substitutional site for Rh, shown as a black dashed line and very close to CO/Rh_1/TiO_{2+2x} where Rh is in supported site), CO/Rh_1@TiO_{2-2x}, CO/Rh_1/TiO_{2-x}, CO/Rh_1/TiO_{2-2x}. b. 2CO adsorption. The most stable site from O rich to O poor conditions is 2CO/Rh_1/TiO_{2+x}, 2CO/Rh_1/TiO_2, 2CO/Rh_1/TiO_{2-2x}.

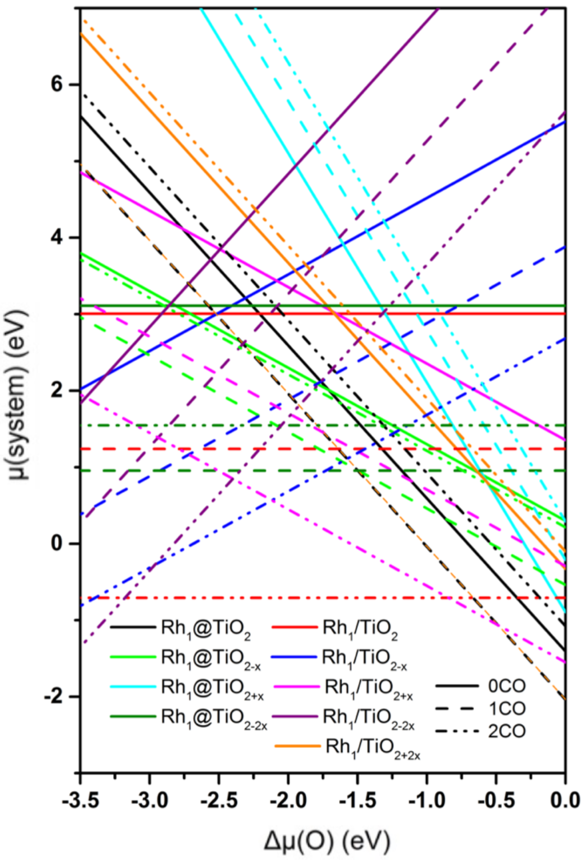

Supplementary Figure 9 | Relative stability of Rh_1TiO_2 structures under a pressure of CO as a function of μ(O). The data represent a horizontal cut of Figure 4 is calculated for a given CO chemical potential (Δμ(CO) = -0.59 eV corresponding to P_{CO}= 0.1 atm and T=300 K). The most stable site from O rich to O poor conditions is CO/Rh_1@TiO_2 (very close to CO/Rh_1/TiO_{2+2x}), 2CO/Rh_1/TiO_{2+x}, 2CO/Rh_1/TiO_2, 2CO/Rh_1/TiO_{2-2x}.

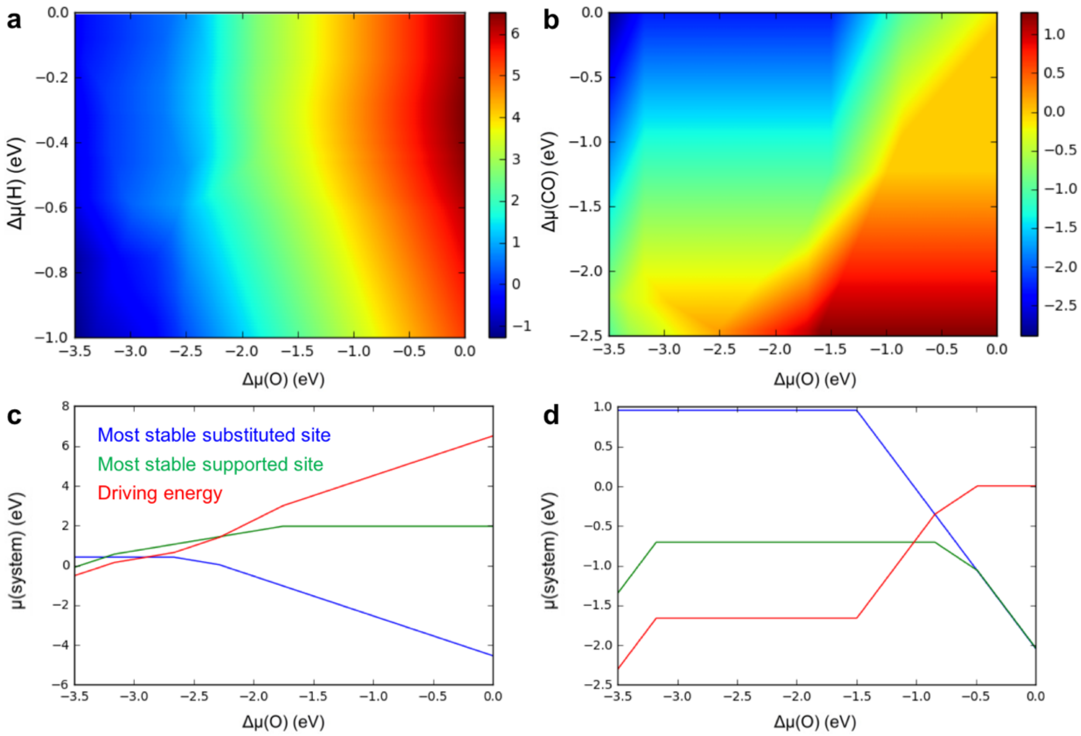

Supplementary Figure 10 | Driving energy for site change of the Rh atom from substituted to supported site (negative value means that site change is thermodynamically favorable). a,c, the calculated driving energy in H_2 reduction. b,d, the calculated driving energy in CO adsorption. The color in a,b represents the value of driving energy. c is a horizontal cut of a at typical condition (H_2 at 10% atmospheric pressure at 500 K, i.e. Δμ(H) = -0.35 eV). d is a horizontal cut of a at typical condition in b (CO at 10% atmospheric pressure at 300 K, i.e. Δμ(CO) = -0.59 eV).

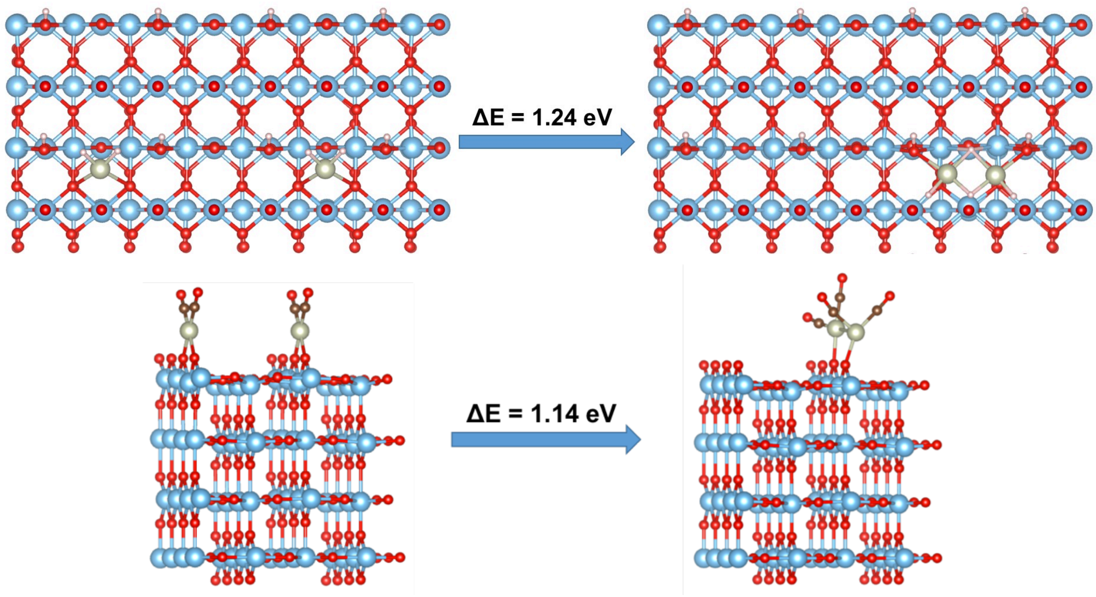

Supplementary Figure 11 | The dimerization of the Rh single atoms is endoenergetic. Top: dimerization of RhH_2 in the conditions of the reduction with hydrogen. Bottom: dimerization of $Rh(CO)_2$ under a pressure of CO. Both reaction energies are endothermic.

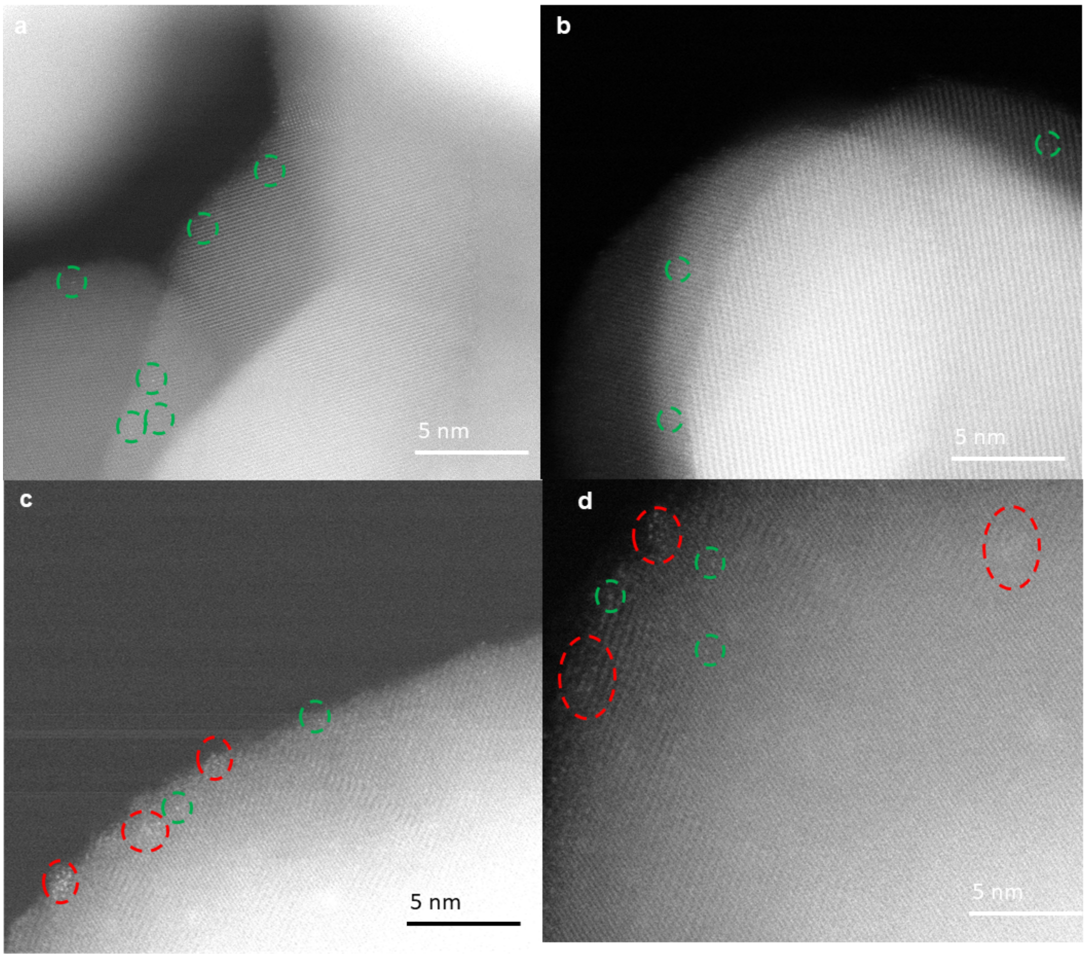

Supplementary Figure 12 | STEM imaging of samples after varying temperature reduction. a, b, Additional HAADF STEM images of 100 °C H_2 reduced sample where only single Rh atoms were observed. c, d, 300 °C H_2 reduced sample in which small Rh clusters and Rh single atoms are observed. Green circles identify the single Rh atoms, while red circles identify Rh clusters.

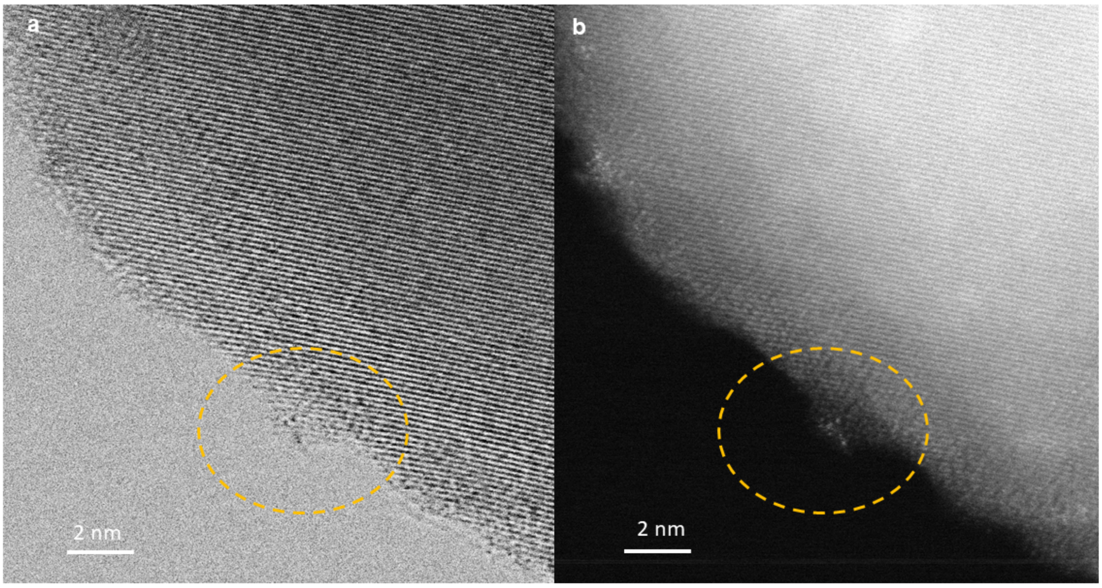

Supplementary Figure 13 | a. 300 °C H_2 reduced sample in which SMSI formation over small Rh clusters can be observed in the Bright Field (BF) image and b. corresponding HAADF image where small Rh cluster can be observed.

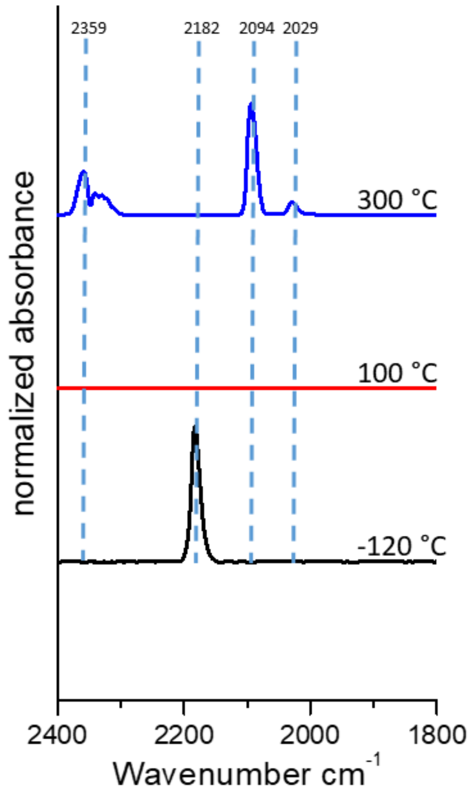

Supplementary Figure 14 | IR spectra of Rh SAC on TiO_2 that had been pre-oxidized at 350 °C and then exposed to CO at -120 °C where CO adsorbs onto Ti^{+4} (evident by 2182 cm^{-1} peak) , 100 °C where no CO is adsorbed, and 300 °C where CO acts as a reducing agent to create oxygen vacancies (evident by CO_2 2359 cm^{-1} peak) so that CO may adsorb onto Rh (evident by 2094 and 2029 cm^{-1} peaks).

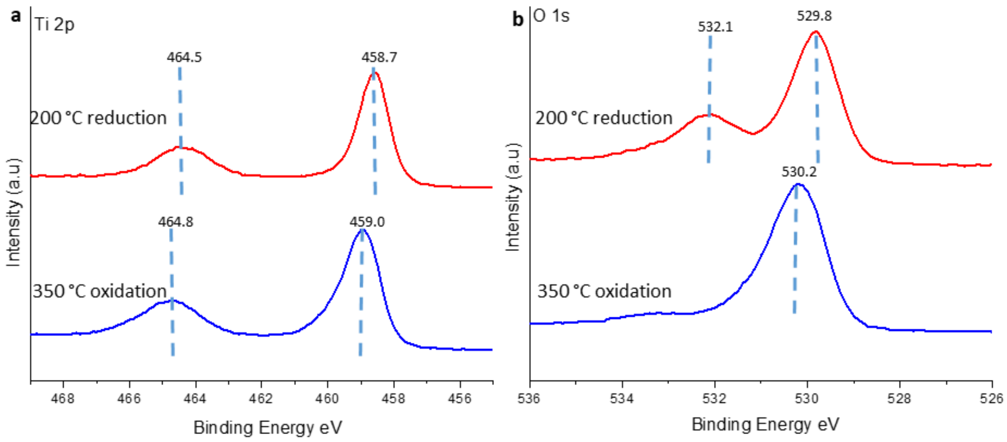

Supplementary Figure 15 | a) XPS spectra in Ti 2*p* region b) XPS spectra in O 1*s* region of Rh SAC on TiO_2 after oxidation in pure O_2 at 350 °C for 30 min and after reduction in 5% H_2 in argon at 200 °C for 1 hour. After both samples were oxidized or reduced, they were vacuum sealed without exposure to air, transferred to a glovebox under inert gas to be mounted in the sample holder and then transferred directly into XPS for analysis.

Supplementary Table 1. Calculated system chemical potential μ(system) for various terminations and positions of the Rh atoms in RWGS reaction conditions (500 K, $10CO_2$:$1H_2$). $\Delta\mu(O)$ is calculated to be -3.03 eV if we assume that the reaction is at chemical equilibrium.

System	μ(system)
$6H/Rh_1@TiO_2$	1.92
$7H/Rh_1@TiO_{2-x}$	1.20
$6H/Rh_1@TiO_{2-2x}$	0.77
$4H/Rh_1/TiO_2$	2.32
$6H/Rh_1/TiO_{2-x}$	1.06
$6H/Rh_1/TiO_{2-2x}$	1.20
$4H/CO/Rh_1/TiO_{2-x}$	0.56

Supplementary discussion

More details to calculate $\Delta\mu(O)$:

1. We define the initial condition: 500 K, 0.1 atm CO_2, 0.01 atm H_2, 0.89 atm He.
2. We calculate the partial pressure when the reaction reaches chemical equilibrium (i.e. $\Delta G = 0$): 0.0999 atm CO_2, 0.0099 atm H_2, 0.0001 atm CO, 0.0001 atm H_2O.
3. We calculate the $\mu(O)$.
   $\mu(O) = \mu(H_2O) - \mu(H_2) = \mu(CO_2) - \mu(CO) = -7.96$ eV, i.e. $\Delta\mu(O) = -3.03$ eV.

Supplementary Table 2. Calculated reaction energies (Δ*E*), Gibbs free energies (ΔG) and energy activation barriers (E_a) for elementary steps in the RWGS reaction on the supported site $6H/Rh_1/TiO_{2-x}$. These elementary steps can be found in Figure 6.

Reaction pathway		Energy		Gibbs Free Energy	
		Δ*E* (eV)	E_a (eV)	Δ*G* (eV)	E_a (eV)
1)	v+6H* → 2v+4H*+H_2O*	0.91	1.05	0.91	1.05
2)	2v+4H*+H_2O* → 2v+4H*+H_2O	0.81	--	-0.49	--
3)	2v+4H*+CO_2 → 2v+4H*+CO_2*	-1.70	--	-0.56	--
4)	2v+4H*+CO_2* → v+4H*+CO*	-0.33	0.26	-0.33	0.26
5)	v+4H*+CO*+H_2 → v+6H*+CO*	-0.34	--	0.47	--
6)	v+6H*+CO* → v+6H*+CO	1.38	--	0.03	--

Supplementary Note 1: The contribution of the entropy term to the free energies of each gas phase is taken from D.R. Stull, H. Propher, JANAF Thermochemical Tables, U. S. National Bureau of Standards, Washington, DC, 1971.

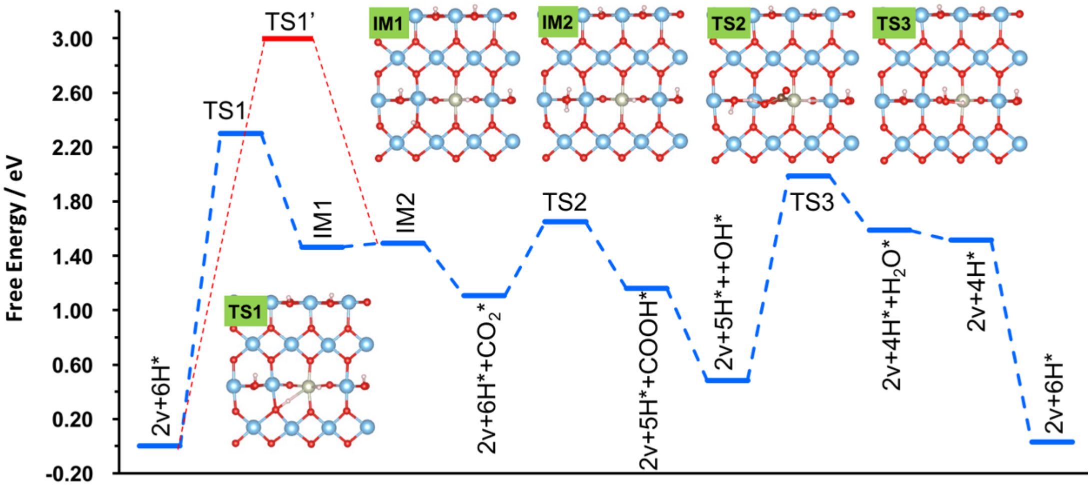

Supplementary Figure 16| Free energy profile of RWGS reaction on the substitutional site 6H/Rh_1@TiO_{2-2x}. Color code: O-red; Ti-blue; Rh-gray. Rh is initially liganded by 2 H atoms. The first step is a H transfer from the Rh atom to surface -OH species to form H_2O, with an endothermic energy of 1.49 eV and a barrier of 2.30 eV. Then CO_2 can adsorb on Rh atom with an adsorption energy of -1.53 eV. The dissociation of the C-O bond via the COOH species is more facile than from the CO_2 molecule directly. After the dissociation of the COOH species, the remaining OH species will react with H atom on Rh atom to form H_2O. The formed water can desorb to gas easily at reaction temperature, and H_2 can adsorb on the Rh atom to reform the initial structure.

Supplementary Table 3. Calculated reaction energies (Δ*E*), Gibbs free energies (ΔG) and energy activation barriers (E_a) for elementary steps in the RWGS reaction on the substitutional site 6H/Rh_1@TiO_{2-2x}. These elementary steps can be found in Supplementary Figure 14.

Reaction pathway	Energy		Gibbs Free Energy	
	Δ*E* (eV)	E_a (eV)	Δ*G* (eV)	E_a (eV)
1) 2v+6H* → IM1	1.46	2.30	1.46	2.30
2) IM1 → IM2	0.03	--	0.03	--
3) IM2+CO_2→ 2v+6H*+CO_2*	-1.53	--	-0.39	--
4) 2v+6H*+CO_2*→2v+5H*+COOH*	0.06	1.16	0.06	1.16
5) 2v+5H*+COOH*→2v+5H*+CO+OH*	0.67	--	-0.68	--
6) 2v+5H*+OH* → 2v+4H*+H_2O*	1.10	1.50	1.10	1.50
7) 2v+4H*+H_2O* → 2v+4H*+H_2O	1.23	--	-0.07	--
8) 2v+4H*+H_2 → 2v+6H*	-2.30	--	-1.48	--

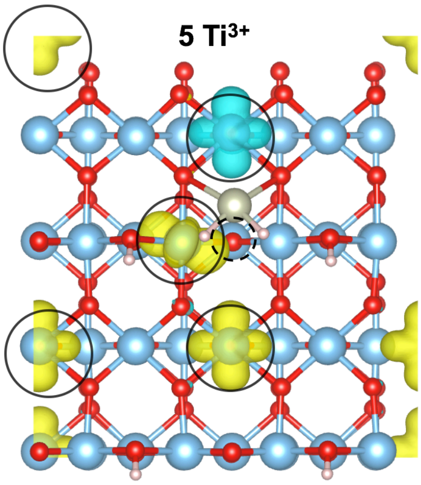

Supplementary Figure 17 | Spin density map for $6H/Rh_1/TiO_{2-x}$ showing the 5 Ti^{3+} centers. Yellow and blue areas represent charge increase and reduction, respectively. Five black circles indicate the Ti^{3+} centers, and one dashed circle indicates the position of O vacancy.

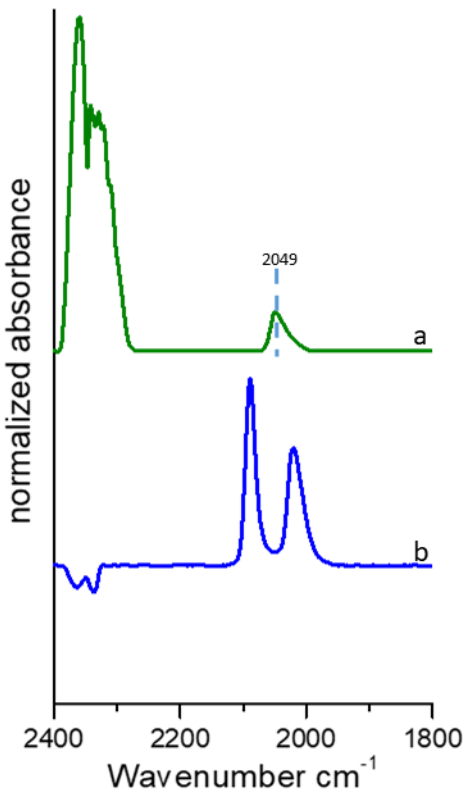

Supplementary Figure 18 | a) In-situ FTIR of Rh SAC on TiO_2 after reduced in 10% H_2 in argon for at 200 °C for 1 hour and exposed to reaction conditions (200 °C, $10CO_2:1H_2$) for 30 minutes. b) Same Rh SAC on TiO_2 after exposed to reaction conditions, purged in argon at 200 °C, cooled for 1 hour in argon to 20° C, exposed to 10% CO in argon for 10 min, and then purged in argon for 10 min.

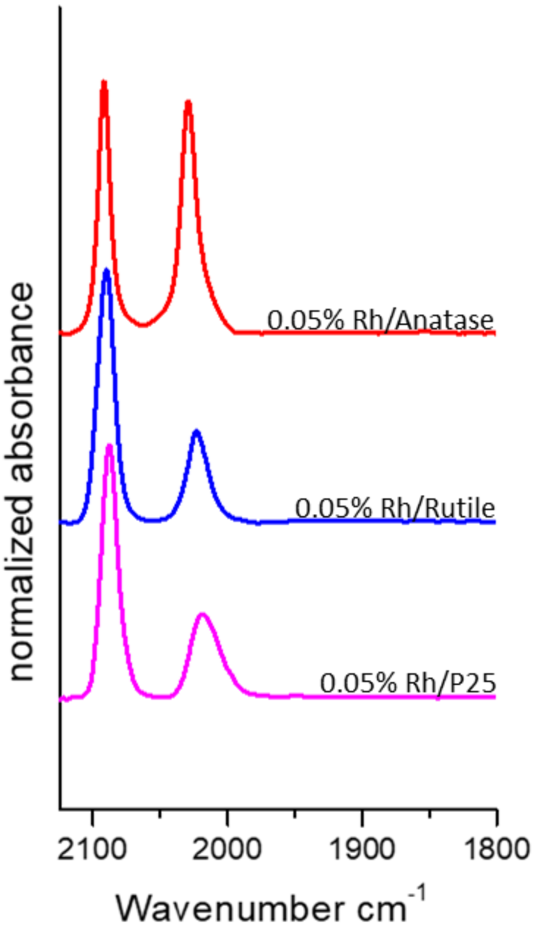

Supplementary Figure 19 | CO probe molecule IR spectra of 0.05% Rh SAC using TiO_2 supports with different phases (Anatase, Rutile, and P25) that have undergone the same synthesis and pretreatment conditions (350 C oxidation and 200 C reduction in H_2) and were then exposed to CO at 20 °C. The ratio of the symmetric stretch to the asymmetric stretch of Rutile and P25 are in close agreement, indicating the same Rh SAC site is identified, whereas the Rh SAC on Anatase creates a ratio closer to 1 and thus constitutes a different Rh SAC active site.

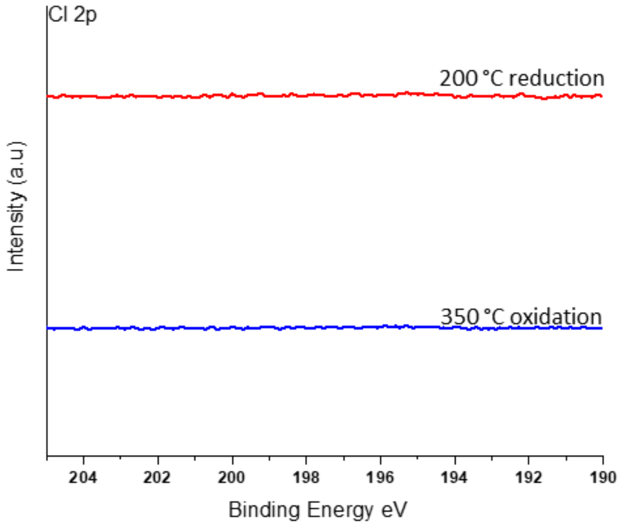

Supplementary Figure 20| XPS spectra in Cl 2p region of Rh SAC on TiO_2 after oxidation in pure O_2 at 350 °C for 30 min and after reduction in 5% H_2 in argon at 200 °C for 1 hour. After both samples were oxidized or reduced, they were vacuum sealed without exposure to air, transferred to a glovebox under inert gas to be mounted in the sample holder and then transferred directly into XPS for analysis.
