# Supplementary material for: Rh single atoms on TiO2 dynamically respond to reaction conditions by adapting their site
Source: Nat Commun. 2019 Oct 3;10:4488. doi: 10.1038/s41467-019-12461-6 (PMC6776542; doi:10.1038/s41467-019-12461-6)
Supplement: Supplementary file 2 — Peer Review File [file 41467_2019_12461_MOESM2_ESM.pdf]

Reviewers' comments:

Reviewer #1 (Remarks to the Author):

This work provided theoretical predictions for the spatial and electronic characters of the singe-Rh site on rutile TiO_2, in response to the variations in reactive atmospheres and temperatures that are relevant to the reverse water-gas shift. In addition, experimental data, mainly IR, partially supported the major conclusions.
It is better to clarify the active sites under reaction conditions (500 K, $10CO_2:1H_2$) they adapted. Here, the powder sample could be routinely analyzed and tested for the target reaction, which would provide direct evidences on the structural properties and reaction rates (or TOFs). A combination of the experimental data with the theoretical simulations will make the conclusions solid. The authors claimed the experimental data in other publications (0.5-2wt.%Rh/P25 (a mixture phase of anatase and rutile), reference 35, JACS 2015), but they are less relevant to the current one (0.05wt.%Rh/TiO_2(rutile)) if the catalytic performance is concerned.

CO-IR analysis confirmed a majority of the Rh species presented as Rh single atoms under the various conditions examined in this work. Upon harsh reduction conditions, single Rh atoms preferentially located above TiO_2 surface. But this would induce aggregation of the metallic atoms as well. As such, STEM analysis would evidence the population of the metal species after H_2 or CO reduction.

Reviewer #2 (Remarks to the Author):

Authors have addressed all my comments except the one "I would like to see the representative STEM images to confirm there is no Rh nanoparticles present after H_2 or CO reduction". I fully understand that rationales of using extremely low Rh loading (0.05%) coupled with CO-IR. However, if all Rh are indeed dispersed atomically, TEM characterization should unambiguously provide Rh atom density on TiO_2 to ensure all Rh atoms are counted by very high confidence. Such information will be critical not only to validate the CO-IR results, but more importantly to support the main conclusions in this paper on Rh single atom dynamics.

Reply to reviewer comments for NCOMMS-19-19076-T

Responses to the referee comments are in **bold** and additions or modifications to the paper are provided in **red**

Reviewers' comments:

Reviewer #1 (Remarks to the Author):

This work provided theoretical predictions for the spatial and electronic characters of the singe-Rh site on rutile TiO2, in response to the variations in reactive atmospheres and temperatures that are relevant to the reverse water-gas shift. In addition, experimental data, mainly IR, partially supported the major conclusions.

It is better to clarify the active sites under reaction conditions (500 K, 10CO2:1H2) they adapted. Here, the powder sample could be routinely analyzed and tested for the target reaction, which would provide direct evidences on the structural properties and reaction rates (or TOFs). A combination of the experimental data with the theoretical simulations will make the conclusions solid. The authors claimed the experimental data in other publications (0.5-2wt.%Rh/P25 (a mixture phase of anatase and rutile), reference 35, JACS 2015), but they are less relevant to the current one (0.05wt.%Rh/TiO2(rutile)) if the catalytic performance is concerned.

The previously reported reactivity results for Rh single atoms on P25 can be compared to the theoretical results here on Rh/rutile TiO2 because the local coordination of Rh species is similar in both samples. We have previously studied atomically dispersed Rh on pure phase anatase, rutile, and mixed phase P25 TiO_2 samples. The CO probe molecule IR spectra of these three identical weight-loading samples are shown in the Figure below. The first noticeable feature is that the symmetric to asymmetric CO stretching bands are of equal intensity for the anatase TiO_2 support, and significantly different for both the P25 and rutile supports. The band shapes and positions for Rh/rutile TiO2 and Rh/P25 are indistinguishable and thus we conclude that Rh single atoms preferentially located on rutile TiO2 domains in the P25 sample that was previously analyzed (JACS 2015). We note that the different in the intensity of the symmetric and asymmetric CO stretching bands was captured by the DFT calculations in the current paper and was primarily due to the existence of O-vacancies at the rutile TiO_2 surface, which does not occur for anatase TiO_2. These results provide strong evidence for the theoretical and experimental analysis of rutile TiO_2 supported single Rh atoms and a comparison of these behaviors with those previously reported for P25 supported single Rh atoms (JACS 2015).

This is addressed in the main text on page 12 and SI with the comment and figure below.

"Comparing the theoretical and experimental results obtained here for rutile TiO_2 supported single atoms with reactivity measurements made on P25 mixed phase TiO_2 supports is justified based on CO IR measurements that suggest single Rh species localized on rutile domains of the P25 TiO_2 support."

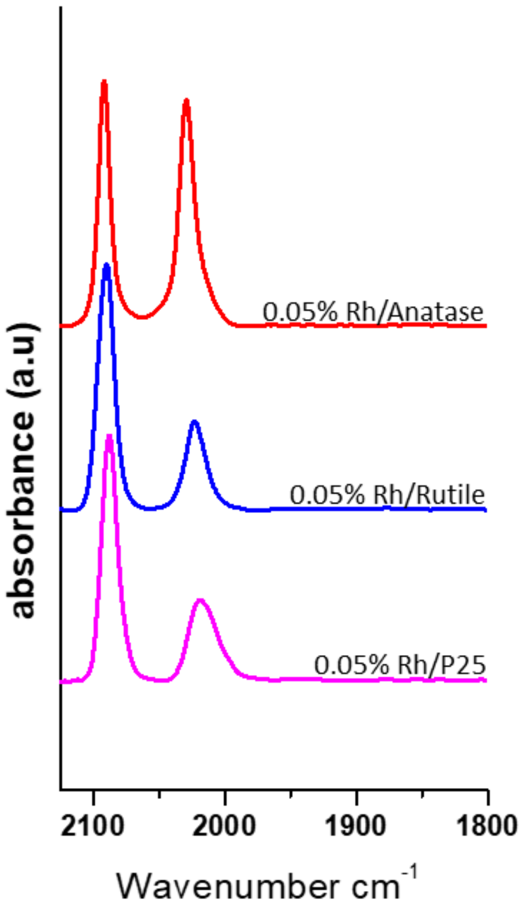

Figure S19 | CO probe molecule IR spectra of 0.05% Rh SAC using TiO_2 supports with different phases (Anatase, Rutile, and P25) that have undergone the same synthesis and pretreatment conditions (350 C oxidation and 200 C reduction in H_2) and were then exposed to CO at 20 °C. The ratio of the symmetric stretch to the asymmetric stretch of Rutile and P25 are in close agreement, indicating the same Rh SAC site is identified, whereas the Rh SAC on Anatase creates a ratio closer to 1 and thus constitutes a different Rh SAC active site.

CO-IR analysis confirmed a majority of the Rh species presented as Rh single atoms under the various conditions examined in this work. Upon harsh reduction conditions, single Rh atoms preferentially located above TiO2 surface. But this would induce aggregation of the metallic atoms as well. As such, STEM analysis would evidence the population of the metal species after H2 or CO reduction.

As requested by the referee we performed STEM imaging of the sample following reduction at 100 C and 300 C in H2. The STEM results are in excellent agreement with the calculations and CO FTIR analysis in that Rh stays a single atoms following 100 C reduction, but becomes mobile and begins to form Rh clusters following 300 C reduction. Thus, the STEM imaging confirms the previous hypothesis. We have added the comment below and images in Figure 5 and S12. Mingjie Xu, George W. Graham and Xiaoqing Pan performed the STEM experiments and have hence been added as authors.

See addition on page 9

"The primary conclusions of the CO probe molecule IR characterization were substantiated via ex-situ atomic resolution STEM imaging of the Rh/TiO_2 sample following reduction in H_2 at 100 and 300 °C, see Figure 5c,d and Figure S12. For the sample that had been reduced at 100 °C, single Rh atoms were primarily observed in all images. Alternatively, for the sample that had been reduced at 300 °C a mixture of Rh clusters and single atoms were observed. Both observations are in agreement with interpretation of the CO IR characterization."

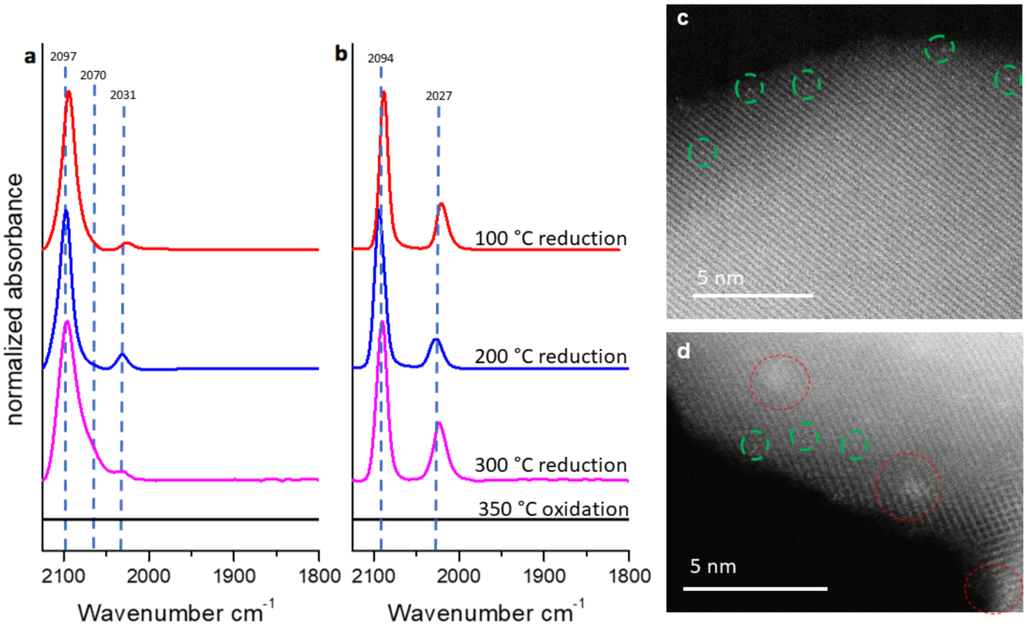

Figure 5 | Experimental analysis of Rh SAC on TiO_2 following varying environmental

treatments. IR spectra of CO adsorbed at full saturation coverage following varied pretreatment conditions (oxidized at 350 °C, reduced at 100 °C, 200 °C and 300 °C) measured at a. -120 °C and b. 20 °C. Corresponding representative high-angle annular dark-field (HAADF) STEM images of c. 100 °C H_2 reduction case in which single Rh atoms are observed and d. 300 °C H_2 reduction case in which small Rh clusters and Rh single atoms are observed. Green circles identify the single Rh atoms, while red circles identify Rh clusters.

Reviewer #2 (Remarks to the Author):

Authors have addressed all my comments except the one "I would like to see the representative STEM images to confirm there is no Rh nanoparticles present after H2 or CO reduction". I fully understand that rationales of using extremely low Rh loading (0.05%) coupled with CO-IR. However, if all Rh are indeed dispersed atomically, TEM characterization should unambiguously provide Rh atom density on TiO2 to ensure all Rh atoms are counted by very high confidence. Such information will be critical not only to validate the CO-IR results, but more importantly to support the main conclusions in this paper on Rh single atom dynamics.

As requested by the referee we performed STEM imaging of the sample following reduction at 100 C and 300 C in H2. The STEM results are in excellent agreement with the calculations and CO FTIR analysis in that Rh stays a single atoms following 100 C reduction, but becomes mobile and begins to form Rh clusters following 300 C reduction. Thus, the STEM imaging confirms the previous hypothesis. We have added the comment below and images in Figure 5 and S12. Mingjie Xu, George W. Graham and Xiaoqing Pan performed the STEM experiments and have hence been added as authors.

See addition on page 9

"The primary conclusions of the CO probe molecule IR characterization were substantiated via ex-situ atomic resolution STEM imaging of the Rh/TiO_2 sample following reduction in H_2 at 100 and 300 °C, see Figure 5c,d and Figure S12. For the sample that had been reduced at 100 °C, single Rh atoms were primarily observed in all images. Alternatively, for the sample that had been reduced at 300 °C a mixture of Rh clusters and single atoms were observed. Both observations are in agreement with interpretation of the CO IR characterization."

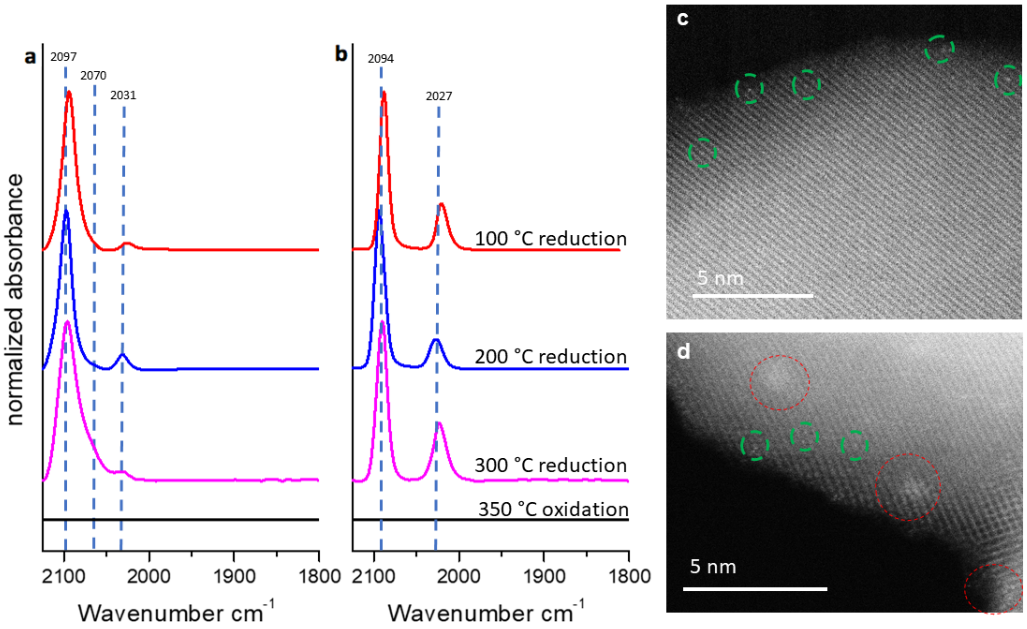

Figure 5 | Experimental analysis of Rh SAC on TiO_2 following varying environmental treatments. IR spectra of CO adsorbed at full saturation coverage following varied pretreatment conditions (oxidized at 350 °C, reduced at 100 °C, 200 °C and 300 °C) measured at a. -120 °C and b. 20 °C. Corresponding representative high-angle annular dark-field (HAADF) STEM images of c. 100 °C H_2 reduction case in which single Rh atoms are observed and d. 300 °C H_2 reduction case in which small Rh clusters and Rh single atoms are observed. Green circles identify the single Rh atoms, while red circles identify Rh clusters.

REVIEWERS' COMMENTS:

Reviewer #1 (Remarks to the Author):

The authors have detailed the questions, raised by the reviewers, with experimental data and reasonable discussion. Overall, the quality of this work has been essentially promoted. I support publication of the current version.

Reviewer #2 (Remarks to the Author):

Authors have adequately addressed my comments.
